# Microbial biomanufacturing for space-exploration—what to take and when to make

**DOI:** 10.1038/s41467-023-37910-1

**Published:** 2023-04-21

**Authors:** Nils J. H. Averesch, Aaron J. Berliner, Shannon N. Nangle, Spencer Zezulka, Gretchen L. Vengerova, Davian Ho, Cameran A. Casale, Benjamin A. E. Lehner, Jessica E. Snyder, Kevin B. Clark, Lewis R. Dartnell, Craig S. Criddle, Adam P. Arkin

**Affiliations:** 1 Center for the Utilization of Biological Engineering in Space (CUBES), Berkeley, CA USA; 2 Department of Civil and Environmental Engineering, Stanford University, Stanford, CA USA; 3 Department of Bioengineering, University of California Berkeley, Berkeley, CA USA; 4 Wyss Institute for Biologically Inspired Engineering at Harvard University, Boston, MA USA; 5 Circe Bioscience Inc., Somerville, MA USA; 6 School of Information, University of California Berkeley, Berkeley, CA USA; 7 Department of Bionanoscience, Delft University of Technology, Delft, South Holland Netherlands; 8 Blue Marble Space Institute of Science, Seattle, WA USA; 9 Cures Within Reach, Chicago, IL USA; 10 Champions Program, eXtreme Science and Engineering Discovery Environment (XSEDE), Urbana, IL USA; 11 Department of Life Sciences, University of Westminster, London, UK

**Keywords:** Biotechnology, Aerospace engineering

## Abstract

As renewed interest in human space-exploration intensifies, a coherent and modernized strategy for mission design and planning has become increasingly crucial. Biotechnology has emerged as a promising approach to increase resilience, flexibility, and efficiency of missions, by virtue of its ability to effectively utilize in situ resources and reclaim resources from waste streams. Here we outline four primary mission-classes on Moon and Mars that drive a staged and accretive biomanufacturing strategy. Each class requires a unique approach to integrate biomanufacturing into the existing mission-architecture and so faces unique challenges in technology development. These challenges stem directly from the resources available in a given mission-class—the degree to which feedstocks are derived from cargo and in situ resources—and the degree to which loop-closure is necessary. As mission duration and distance from Earth increase, the benefits of specialized, sustainable biomanufacturing processes also increase. Consequentially, we define specific design-scenarios and quantify the usefulness of in-space biomanufacturing, to guide techno-economics of space-missions. Especially materials emerged as a potentially pivotal target for biomanufacturing with large impact on up-mass cost. Subsequently, we outline the processes needed for development, testing, and deployment of requisite technologies. As space-related technology development often does, these advancements are likely to have profound implications for the creation of a resilient circular bioeconomy on Earth.

## Abstract (web summary)

Biotechnology is emerging as a promising approach to increase resilience, flexibility, and efficiency of space missions. In this Perspective, the authors outline design-scenarios and provide a techno-economic analysis of their deployment.

## Introduction and background

With reinvigorated curiosity and enthusiasm for space-exploration and increasingly complex campaigns, humanity prepares to return to the Moon en route to Mars^1–3^. Efforts to modernize mission architectures^4,5^—combinations of inter-linked system elements that synergize to realize mission goals^6^—will need to leverage an array of enabling technologies including biomanufacturing towards the realization of such grand visions^7–9^. Microbial biomanufacturing has the potential to provide integrated solutions for remote or austere locations, especially where supply chains for consumable and durable goods cannot operate reliably^10,11^. Complementary to, but distinguished from merely remediative and extractive microbial functions, such as biomining^12,13^, off-world biomanufacturing corresponds to any deployable system that leverages biology as the primary driver in generating mission-critical inventory items of increased complexity, i.e., the de novo synthesis of components for the formulation of food, pharmaceuticals, and materials^8,10,14,15^. When integrated effectively into mission architectures, bio-based processes could significantly de-risk crewed operations through increased autonomy, sustainability, and resilience, freeing up payload capacity^16^.

Key to the efficacy of biotechnology as a support of human space-exploration is its efficiency in using locally available resources (in situ resource utilization, ISRU) and the ability to utilize waste streams from other mission elements and recycle its own products (loop-closure, LC)^17–19^. As missions expand, progressive advancement and wider implementation of in situ (bio)manufacturing (ISM/bio-ISM) will lead to greater independence, enabling more complex mission-designs with extended goals, and may eventually allow a self-sufficient human presence across the solar system to be sustained. Biomanufacturing is appropriate for that purpose, because high-volume resources, like fixed carbon and nitrogen (as well as low-volume, but critical resources such as minerals) can be produced and recovered in compact autonomous systems that are analogous to Earth’s biogeochemical cycles^20–24^. Biochemistry also provides access to a plethora of organic compounds, often at unrivaled purity and selectivity, many of which are not accessible by other means^25,26^.

**Fig. 1:**
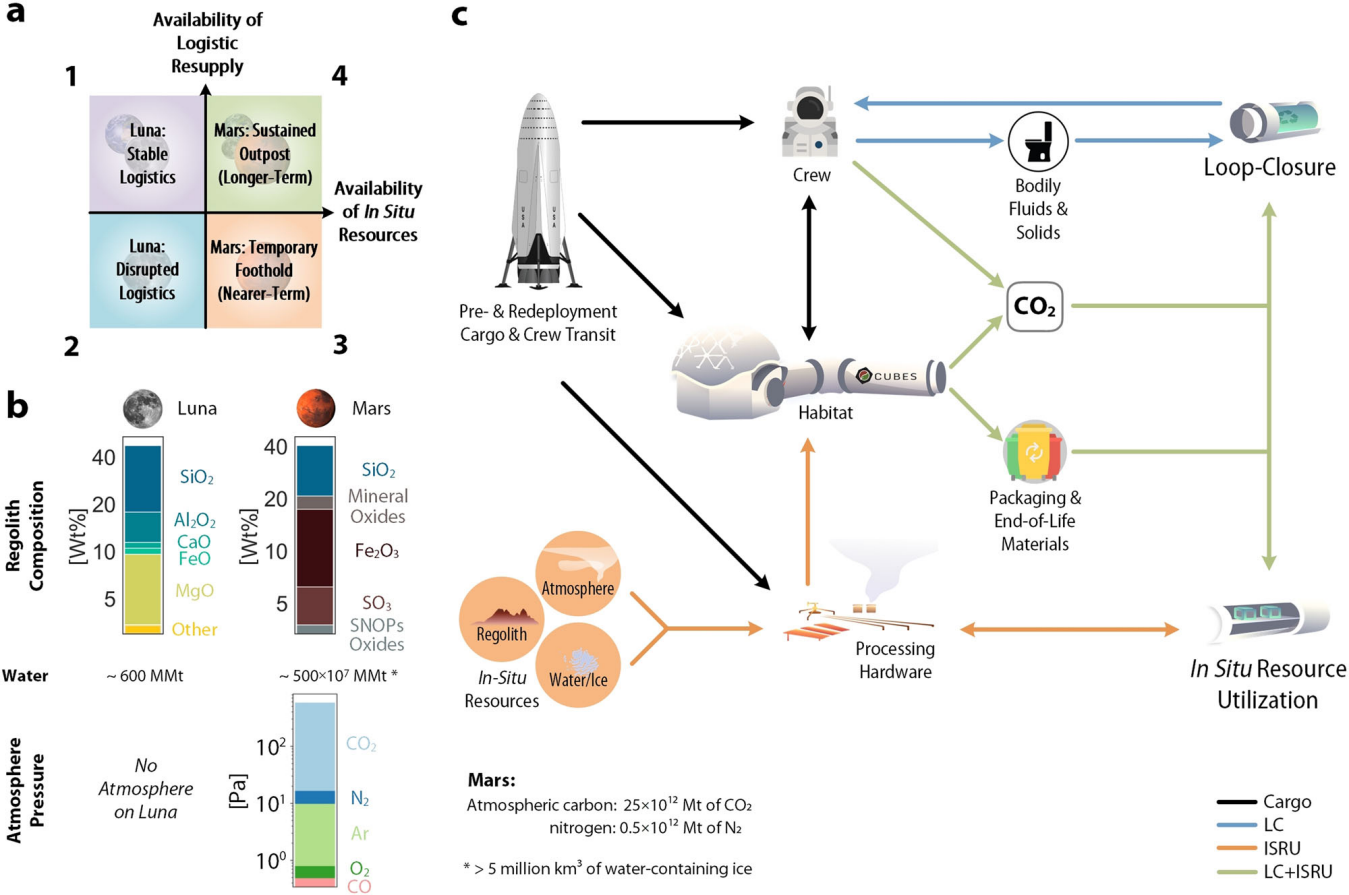
Approaches to in situ biomanufacturing (bio-ISM) depending on off-world mission-class. The context-specific off-world mission-classes 1 to 4 are defined in (**a**), mapped as quadrants on qualitative spectra for the availability of in situ resources and logistic resupply. The most ubiquitous surface-accessible in situ resources for the Moon^101^ and Mars^102^ are compared in (**b**) in form of gases and solids, broken down into their molecular compositions (SNOPs: sulfur, nitrogen, oxygen, phosphorus. Note that SNOPs and mineral oxides exclude otherwise listed compounds. Compositions and amounts (where given) are rough estimates, based on current knowledge^103–106^. MMt: million metric tons). Biomanufacturing concepts-of-operations (CONOPS), outlined in (**c**), are color-coded for the operational mode: outgoing from initial cargo (black lines), CONOPS can rely on either loop-closure (LC, blue lines), in situ resource utilization (ISRU, orange lines), or both (LC+ISRU, green lines).

Biologically-driven ISM in support of space-exploration becomes more significant the deeper humans venture into space: As the support of supply chains becomes increasingly challenging the further humans travel, ISM is most feasible in locations where resources are available, accessible and abundant, such as the Moon^27^, but even more so Mars (Fig. 1b). The advantages and drawbacks of biotic and abiotic approaches for ISM, in particular for life-support but also auxiliary functions for extended human operations beyond Earth-orbit have previously been discussed at length^8,10,28,29^ (also see Table 1 in the Supplmentary Information), but an actionable roadmap for deploying biomanufacturing-based systems within upcoming campaigns has yet to be formulated. Here, we discuss the applicability of biologically driven ISRU and LC in the context of different off-world mission classes, and conduct a qualitative techno-economic analysis (TEA) to unravel the inventories of different mission-design scenarios of space-travel. Following the TEA, we lay out paths for readying bio-based technologies for deployment and inclusion into mission-architecture, to enable the next phase of roadmapping for crewed missions into deep-space.

## Off-world biomanufacturing approaches

### Concepts-of-operations: modes of in situ bio-manufacturing

Given that biomanufacturing is uniquely suited to play significant roles in the specific realms of food, therapeutics, and materials^8^, a key challenge in realizing its potential rests in the availability and abundance of mobilizable feedstocks^10^—provided through logistic resupply (directly or from re- and up-cycling of mission products) or obtained from in situ resources. This abundance depends on the destination, and leads to a qualitative discrimination of off-world mission-classes as shown in Fig. 1a. The in situ resources that are available on the Moon and Mars are broken down in Fig. 1b, which aids in comparing mission profiles and the extent to which these can be driven by biomanufacturing. For each mission-class, different concepts-of-operations (CONOPS) are applicable—these CONOPS conform to specific inventory needs as they relate to mission- and crew-requirements and depend on the resources availability for ISM. The environmental context informs the specification of feedstocks and processing pipelines (LC or ISRU) for ISM, as shown in Fig. 1c.

Each mission-class comprises a unique set of inventory items; these may include infrastructure components (e.g., habitat assemblies and furnishing, functional hardware/appliances as well as scientific equipment and tools), transported as either pre-deployment cargo or with the crew. These components are used to assemble the larger integrated habitation and life-support systems as well as (bio-)ISM-based LC or ISRU systems and infrastructure related to mission-objectives^30^. While all such off-world mission-classes are distinct in terms of operations^4^, they serve as exemplars to better understand biomanufacturing strategies in relationship to mission-specific factors that might provide resources, crew count/needs, and logistical constraints.

### Implementation of bio-ISM dependent on off-world mission-class

Class 1 (Moon, stable logistics) considers Artemis-like Lunar operations^1^, specifically short stay missions for small numbers of astronauts with narrow scope of scientific and technical exploration. Because of the short times and logistic accessibility, crew-needs for food, medicine and materials can be provided through carry-along and resupply from Earth^31,32^, rather than relying on more complex, risky and time-intensive technologies such as biomanufacturing. Also, due to the dearth of in situ resources on the Moon (Fig. 1b)^27,33^, the scale of biomanufacturing will be constrained by the supply chain and capability for recycling and LC. However, because of the well-supported environment, it is an ideal location to prove and improve technologies for biomanufacturing in space by testing automated and scaling operation of critical bioreactor systems for different bioprocess types (e.g., electro- and photo-autotrophic (gas) bioreactors for lithoautotrophic and/or saprotrophic fermentation of macronutrients^34^), all of which are likely to have physiological and operational challenges in a low-gravity, high-stress environment^35–37^. To this end, systems that have achieved a Technology Readiness Level (TRL)^38,39^ of 5 are well suited to be implemented and evaluated. While these systems currently exist in isolation or partially integrated in laboratory and industrial contexts, building automated end-to-end, compact systems (advancement past TRL 7) will be a key requirement for class 1, so as to meaningfully scale to future, more constrained mission architectures.

Class 2 (Moon, disrupted logistics) considers advanced Lunar operation capabilities when extensive infrastructure has been deployed on the Moon. To increase the time for operations between resupply (and brace against unexpected disruption), storage facilities are increased and biomanufacturing becomes more attractive. Given the paucity of feedstock raw materials on the Moon (central resources necessary for bio-ISM such as carbon and nitrogen are hardly present, water is poorly accessible and distribution highly heterogeneous)^27,33^, hyper-efficient use of stored supplies and efficient use of other available mission products and waste-streams via LC must be engineered. Derivatization of packaging materials, such as biodegradable plastics, and minimal processing systems for black and grey water could provide significant augmentations to expected feedstock and extend the operational times of biomanufacturing systems in the event of scheduled or unplanned disruption of the supply chain^40^. Under extreme conditions, being able to switch biomanufacturing operations from, for example, complex vegetable foods to faster and less resource-intense production of simple cellular foods becomes paramount to defray risk. These challenges require innovations in new alternative feedstock engineering in organisms; co-design of mission materials for biological consumption; development of basic waste-processing systems; and flexible re-configurable infrastructures for production to respond to changing resource conditions. Applicable technologies comprise systems that have been tested in the relevant environments and brought to TRL over 7 within operations of class 1, and are ready for implementation into mission architecture.

Class 3 (Mars, rudimentary logistics) considers basic biomanufacturing systems deployed on Mars with poor logistic resupply due to increased interplanetary distance but with greater availability of in situ resources as compared to the Moon. While mission-design is still characterized by small crews on round-trips, resource constraints carry different weight. Given the extent and degree of the unknowns involved, these missions are ideally designed to maximize safety and stability by preparing for diverse contingencies. Providing those redundancies becomes exceedingly challenging due to the remoteness of Mars^4^. Hence, meaningful bio-ISM is necessary – with substantial scaling of the systems brought to TRL 8 to 9, supported by developments from classes 1 and 2. While a portion of the food, therapeutics and materials will still derive from cargo, significant ISRU of regolith, water, and atmosphere must be implemented in addition to LC, to ensure mission flexibility and resilience. For food, nutritional completeness and palatability, together with customization of texture, flavor, and format will be of central importance. To further safeguard crew-health, essential therapeutics that cannot be included in cargo due to restrictions such as shelf-life, are within scope^8,10,15^. For maximum fidelity of mission operations, a range of thermoplastic multi-purpose materials have been proposed for manufacturing of e.g., food processing equipment, surgical and medical supplies, radiation shielding, and habitat components^41–44^. Enabling technologies include: modular fermentations and bioprocesses at scale, optimized genetically engineered microbial strains to efficiently produce intermediates (i.e., ingredients, agents, crude polymer), and formulation/processing systems to assemble the final products (i.e., meals, drugs, manufactured items). Automation of operations becomes the backdrop of an increasingly diverse set of inventory items.

Class 4 (Mars, developed logistics) envisages a fully fledged and integrated biomanufactory where essential logistic resupply is enabled by interplanetary networks and deep-space outposts^45–47^, combined with extensive ISRU and LC. Specifically, this class would entail sustained human operations on Mars on the verge of permanent settlement. The extensive infrastructure that must be deployed for this kind of mission-design enables production of complete and diverse foods with a spectrum of forms and nutrition, a holistic range of therapeutics, and different bulk as well as specialty materials (plastics, metals, composites) that allow not only the maintenance but also expansion of infrastructure, semi- or fully autonomously. Biomanufacturing technologies and auxiliary infrastructure need to be fully developed and matured to readily deploy tailored microbial cell factories that can potentially be engineered on-demand as the need arises. To this end, even the accommodation of a “space biofoundry” (i.e., automated infrastructure for engineering and analytics of biological systems^48^) in the mission architecture is within scope. Eventually, this will also entail the ISM of specialty chemicals and reagents like e.g., phosphoramidites for DNA synthesis, supporting on-site bioengineering^49^, in addition to the total inventories of foods, therapeutics and materials.

As outlined, depending on the off-world mission-class, biomanufacturing can either be tested and developed (class 1) or used as a mission support tool (class 2 to 4). However, before plans for implementation of biomanufacturing in mission architecture can be put into action, a holistic analysis that can directly compare the trade-offs between different mission-design scenarios with and without biomanufacturing is needed. An initial effort towards such an analysis is presented in the following.

### Off-world mission-scenarios and bio-available inventories

**Fig. 2:**
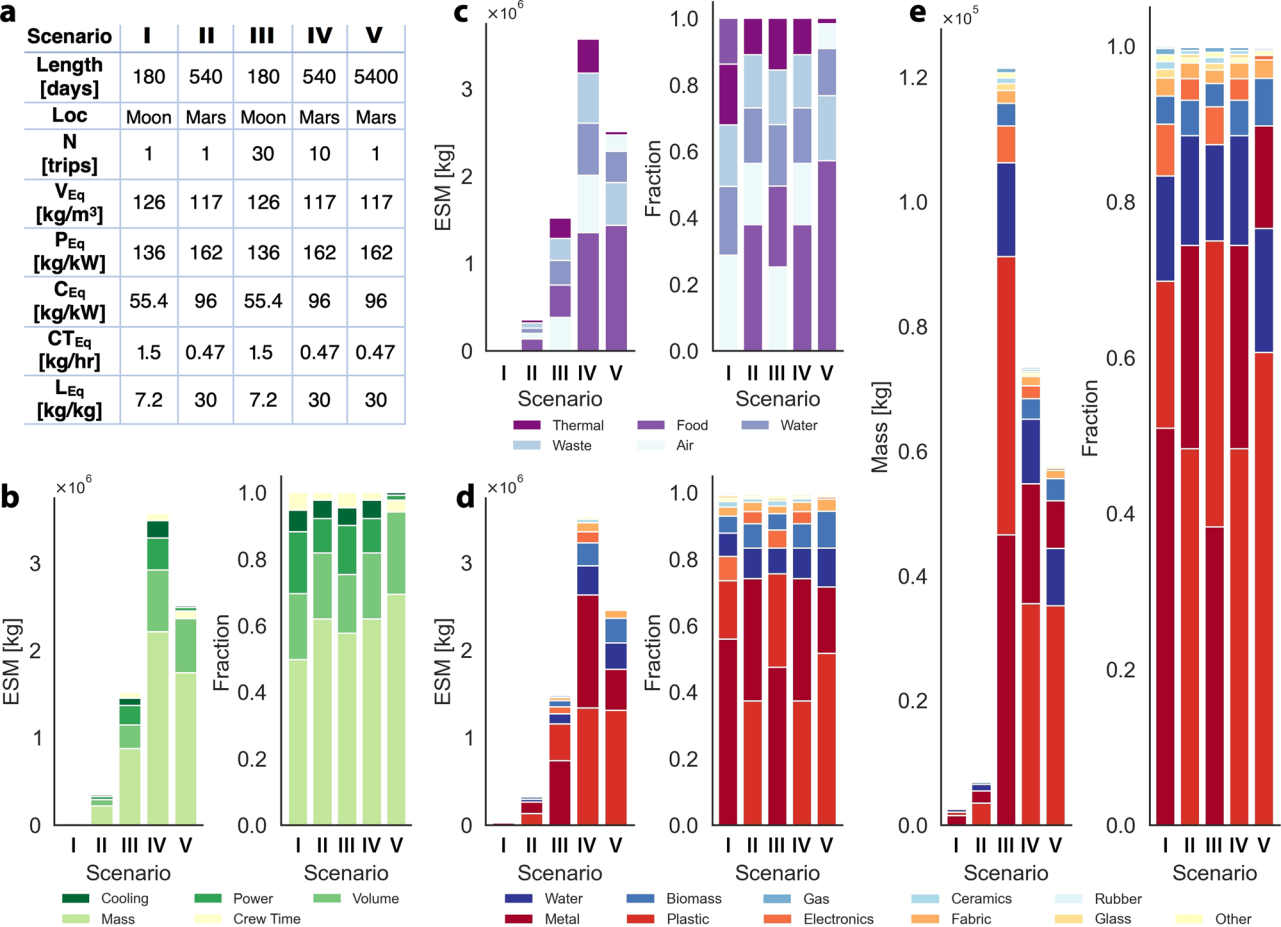
Breakdown of environmental control and life support systems into components by type of system and composition thereof. The make-up of the inventory and hence the operational expenses are dependent on the mission-design scenario. Panel (**a**) provides an overview of parameters for five exemplar space-travel scenarios: ‘I’ and ‘II’ correspond to single sorties (*N*) to the Moon and Mars, respectively, using standard surface operation duration^40^, while ‘III’ and ‘IV’ correspond to multi-sortie campaigns with the same 5400 days of total surface operation as in ‘V’. Based on these parameters and equivalency factors for Volume (*V*_eq_), Power (*P*_eq_), Cooling (*C*_eq_), Crew-Time (*CT*_eq_), and Location (*L*_eq_) the ESM can be calculated for each scenario (as per Eq. 1 in section 2 of the SI)^97^. Panels (**b**–**e**) visualize the inventory breakdown by the expense-type contributing to the total ESM (**b**), type of system-component classified by associated resource (**c**), and composition of the inventory item (**d** and **e**): the bar-charts in panels (**b**–**d**) show the breakdown in ESM units (on the left, in mass [kg]), and the fractional breakdown of each scenario (on the right, unit-less), while in panel (**e**) the absolute (left, in mass [kg]) and fractional (right, unit-less) inventory breakdown in terms of material composition is visualized. An alternative representation of the data presented in (**d** and **e**) is given in Fig. S1c. ESM equivalent systems mass—for more information see BOX 1.

CONOPS for ISM—the flow of resources and integration of LC with ISRU—not only differ depending on the off-world mission-class, but are dependent on and influenced by mission-design scenario. To assess the potential impact that biomanufacturing can have on mission-design more quantitatively, five distinct but comparable scenarios were established as per Fig. 2a (also see Table S2 and Fig. S1a). The outlined scenarios were designed with the objective of greatest comparability among destinations (Moon or Mars) and as such are agnostic of the previously discussed off-world classes which differentiate two levels of technology development for each destination (Fig. 1). Mission-design scenarios ‘I’ and ‘II’ correspond to single sorties to the Moon and to Mars, respectively, using standard surface operation duration^40^. Meanwhile, scenarios ‘III’ to ‘V’ consider 5400 days of surface operations either as multi-sortie campaigns (scenarios ‘III’ and ‘IV’) or in a single sortie (scenario ‘V’). Mission-design scenarios ‘I’ and ‘III’ may correspond to off-world mission-class 1 (or 2), while mission-design scenarios ‘II’, ‘IV’, and ‘V’ correspond to off-world mission-class 3 (or 4).

Using NASA’s ‘Advanced Life Support Sizing Analysis Tool’ (ALSSAT)^50^, an analysis of cargo inventory (environmental control and life support systems) broken down for each mission-design scenario and compared by means of Equivalent Systems Mass (ESM, further information in BOX 1)^51,52^ was conducted (see Supplementary Information section 2 for details on data aggregation and representation). The bar-charts in Fig. 2 decompose the operational expense by means of ESM, differentiated by the term contributing to the total ESM (mass, power, cooling, volume, crew-time in 2b), type of system-component classified by associated resource (waste, food, water, air, thermal in 2c), and composition of the inventory items (structural metal, plastic, water, biomass, electronics, etc. in 2d) for each scenario. This preliminary TEA-data serves as *prima facie* estimation for assessment of mission-cost and provides a primary step towards drawing a relationship from the availability of cargo resources to potential inventory items that lend themselves to ISM, in particular by means of biomanufacturing (Additional details on the compilation of the preliminary TEA-data are provided in both sections 2 and 3 of the SI).

Apart from the realization that long-duration and -distance journeys have the highest ESM effort, it is also not surprising that multiple trips are more expensive than a single sortie. Further, Fig. 2b highlights that across all scenarios the primary expense (in form of ESM) will be mass itself, followed by volume. More importantly, the analysis provides insight into resource and inventory differences, which has implications for applicability of ISM among the different mission-design scenarios: Fig. 2c shows that scenarios ‘I’ and ‘III’ (Moon) are dominated by air systems (~30% and 26%, respectively) while scenarios ‘II’, ‘IV’ and ‘V’ (Mars) are dominated by food systems (~38%, 38% and 59%, respectively), which supports the biomanufactuing schema breakdown in Fig. 1. Further, with an estimated ~12% to 16% of the total cargo-mass being water, the associated systems contribute ~15% to 20% of total ESM (Fig. 2c). Because the mass contribution from water systems is higher for scenarios ‘I’ and ‘II’, any employed biomanufacturing strategy should be geared towards LC for water-recovery and -reuse. The same is true for air supply and conditioning in case of scenarios ‘I’ and ‘III’. Across all scenarios the greatest expenses in form of commodities (2d and 2e) are accountable to structural metals, plastics, water, and biomass. Most notably, Fig. 2c shows that both, the mass and ESM for each scenario, are dominated by cargo composed of metal and plastics. Unfortunately, structural metal is likely to remain unsuitable for biomanufacturing for the foreseeable future. While biomanufacturing for space-exploration is most frequently conceived towards food production or therapeutics for sustaining astronauts, we find that materials hold greatest promise to save up-mass through ISM: in Lunar scenarios, plastic increases from ~19% to ~37% of total mass with increased mission-duration; in Martian scenarios, plastic increases from ~48% to ~60%. This supports the emphasis on ISM of these materials with increasing mission-duration and -reach.

Box 1 Assessment of Economics, feasibility and risk of mission-architecture In-space biomanufacturing systems will need to demonstrate superiority over traditional systems in supporting crewed space-missions. To this end, traditional mission-designs must be directly comparable to those augmented with biomanufacturing. One of the more widely used metric to quantify specific attributes of life-support systems is Equivalent Systems Mass (ESM)^51,52^. In brief, ESM allows mass, volume, power and crew-time to be converted into a single metric in kilograms-equivalent to predict the up-mass requirement as proxy for expense^97^. ESM has become a standard metric also for comparing biomanufacturing systems^98,99^, however, it cannot account for aspects such as risk, sustainability, recyclability, complexity, modularity, reliability, robustness, resilience, readiness, scalability, or safety.

As a complement to ESM, the concept of payback time (PBT)^100^ has been developed to assess some of these criteria – PBT reflects cost, recyclability, and economic sustainability. Specifically, the PBT is useful in assessing ISRU options, as it allows comparison of the cost to launch and deploy (bio)manufacturing capabilities with the cost of a continuous resupply from Earth over time. Adding statistical risk assessments to the PBT can also help to quantify risk, safeguarding robust and reliable systems. For example, the concept could determine the statistical risk of landing on Mars, with the risk reduction of reduced number of landings on one side but a loss of the payloads carrying ISM hardware being more critical than failure of resupplying missions on the other side. The statistical value of those risk-factors must be carefully assessed based on previous missions, the general technology development roadmap, and the expected learning rates on those factors. Through reliable and generalizable analyses like these, the biomanufacturing approaches which are most vital can be meaningfully assessed.

## Integrating biomanufacturing with mission-architecture

### Production of materials for manufacturing

Just like for production food or pharmaceuticals, biomanufacturing of materials may support the fabrication of consumable and durable goods made of plastics^15,53,54^, metals^55,56^, and ceramics^57^ (~18% to 60%, ~13% to 50%, and ~1% of total mission ESM respectively, Fig. 2d). The capacity for off-world ISM of materials and fabrication of mission objects will depend on their uses and sizes ranging from small replacement parts and functional tools to physical components of the life-supporting habitat^58,59^: the extent of initially required critical infrastructure^60,61^ dictates the degree to which ISM can be integrated with mission-architecture.

In combination with additive manufacturing^62^, thermoplastic biomaterials (e.g. polyesters like polyhydroxyalkanoates) could make up the majority of high-turnover items with regular demand, while also providing for contingencies, i.e., non-anticipated servicing and repairs of incidental nature. Such polymeric constructs can be derived from basic feedstocks in a more compact and integrated way when using a bioprocess rather than chemical synthesis. This advantage is greatest in the case of comparatively (to Earth-based manufacturing industry) low demand and throughput^8,10^. Biomanufacturing may also enable the production of high-performance polymers, such as for example thermoset aramids and arylates, which have a range of applications in space technology^53,63^.

While present in abundance, soil and rock are little versatile, due to the limited mechanical properties of regolith (i.e. low flexibility and plasticity)^64–66^. For surface operations as components of buildings and structures, processing via e.g. solar/laser or microwave sintering^67^, and autonomous 3D-printing of regolith composites with binding resins has been proposed and prototyped as means to manufacture infrastructure (see Supplementary Information section 4 for an overview of recent advancements)^68,69^. Both approaches still require significant up-mass to deploy auxiliary equipment for e.g., stripping and processing of topsoil, as well as the raw material for the binding resin. If, however, binding agents (e.g. bioderived thermoplastics or thermosets) could also be produced on-site from in situ resources, an additive manufacturing method may become immediately more feasible^70,71^.

Another possibility to leverage regolith as an in situ resource is to extract certain elements of interest for further processing and application. Specifically, this pertains to metals, the second most contributor to total ESM, as per the forgone analysis (Fig. 2d and e). Performed with microorganisms, a process known as bioleaching or biomining is already being applied on Earth (e.g., for 20% to 30% of global copper production^72^). For space applications, biomining/-leaching is distinguished into three classes of resources: (1) metals and minerals like iron and sulfur oxides^73,74^ or silicates^75^, all of which are common in various regolith types (Fig. 1b) and can be extracted for construction purposes and other bulk applications^13^; (2) rare earth elements for specialty applications like lanthanides, scandium, and yttrium, which can be extracted from specific regolith types^76^; (3) noble metals found in components of electronics brought from Earth (e.g., copper), to be reused for new circuitry. While (1) and (2) are part of ISRU and (3) contributes to LC, all of these extractive bioprocesses can be coupled with additive manufacturing technologies (e.g. Selective Laser Melting & Sintering) for perpetual or on-demand ISM.

Technologies for production of biomaterials, in particular bioplastics, from waste-derived feedstocks such as end-of-life products and/or carbon dioxide are immediately relevant to providing solutions for the most pressing challenges of humanity on Earth. This includes mitigation of greenhouse gas emissions through carbon-capture and -neutrality (i.e., LC), as well as reduction of environmental pollution by replacing durable synthetic materials with biodegradable alternatives. Biomaterials production from inorganic carbon and/or waste-streams is therefore an enabling technology for the evolution of a circular economy and sustainable (bio)chemical industry on Earth^77^. Biomining and -leaching technologies would further contribute to advancement of remediation techniques, also contributing to move towards a more sustainable and circular economy.

### Rational coupling of biological systems and resources

Selection of the specific feedstocks utilizable for different ISM purposes must be guided by critical consideration for recycling of resources at molecular and elemental level—any dead-end, non-recyclable stream will eventually require a resupply from Earth. For auxiliary functions (e.g., materials for additive manufacturing), production volume is more important than continuity and response time (as is critical for food and therapeutics), therefore requiring the adaptation of widely available resources (carbon dioxide and derivable single-carbon compounds or crude biomass)^14,78^, either directly (where available), or through (physico)chemical means (e.g., as secondary beneficiary of propellant production from in situ resources)^79^. Hence, the collective approach to more deeply developing synthetic biological tools for bio-ISM must begin with the feedstocks—sugars or other purified multi-carbon compounds (e.g., higher alcohols and fatty acids) will likely not be the prime substrates of biomanufacturing in space, but rather the products/intermediates in a manufacturing chain or loop that serves life-support (within LC elements such as regeneration of oxygen and waste reclamation)^34^. This includes hybrid-strategies like the abiotic synthesis of sugars as substrates^80^.

**Fig. 3:**
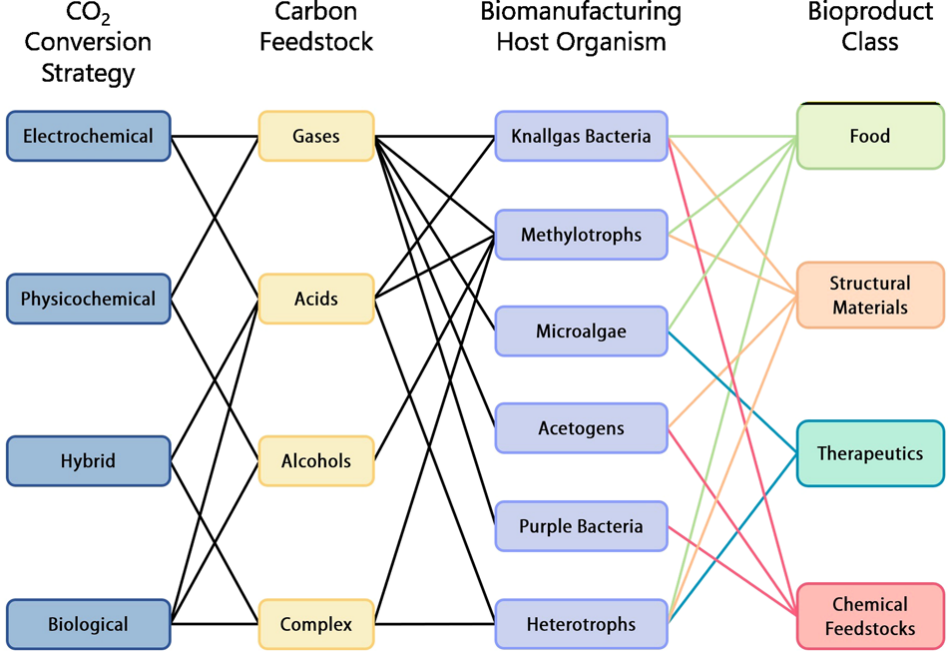
Breakdown of available routes for bioproduction of inventory items from carbon dioxide—either as in situ or recovered resource. Connecting lines represent possible paths for carbon-compound conversion of intermediates to products. Usability of different feedstocks is tied to nutritional mode of the microbial host organism (more than one nutritional mode is possible for certain organisms). Classes of products are assigned to respective microbes in respect of their metabolism as well as not represented ‘shadow-characteristics’ of the chassis (e.g., aerobic/anaerobic, prokaryotic/eukaryotic, metabolic rate, robustness, etc.), rather than ability to (naturally) derive the respective compounds. Products may or may not comprise some of the initial feedstocks, hence consecutive runs through this chart to up-cycle carbon are conceivable.

Critically, because in space savings on payload supersedes commercial relevance, adaptation of non-model microbes that save mass is much more valuable. The range of microbial taxa being proposed and investigated for in space-applications is, however, still narrow and often limited to the few model organisms (e.g., E. coli and *S. cerevisiae*) whose popularity in Earth-based applications is mostly rooted in legacy. Although a great deal of progress has been made to adapt these organisms to utilization of single-carbon feedstocks^81,82^, they are still outclassed by organisms naturally capable of these functions^83–85^. Therefore, species with nutritional modes and metabolism uniquely suited to leverage resources available through LC and ISRU must be considered for development of ISM systems, basing their selection on application (feedstock/product pairing, scale, continuity, and responsiveness of the respective process) and scenario-specific criteria (environmental parameters)^34,86,87^. Specifically, organisms with the ability to assimilate single-carbon compounds alongside organics (mixotrophy) are most suitable. For this purpose, expansion of metabolic engineering efforts to create (synthetic) pathways that increase the carbon-efficiency of metabolism and/or allow the catabolism and subsequent up-cycling of non-natural feedstocks, like e.g., synthetic plastics, is also sensible^88,89^. To illustrate these considerations, a qualitative breakdown of possible production routes/flow of carbon through different biomanufacturing approaches for inventory items from off-world class-dependent in situ resources is established in Fig. 3. While metabolic engineering theoretically allows almost any bio-available compound to be produced in any organism, the effort required for realization can be excessive. For example, oxygen-dependent pathways will hardly be functional in obligate anaerobes without extensive modifications. Likewise, correct folding of proteins with high post-translational modifications in prokaryotes is unlikely.

## Implementing off-world biomanufacturing—fundamental needs

### Readying microbial production systems for space

While the concept of space biomanufacturing has been around for several decades^90–92^, its application is still limited to individual small-scale microgravity experiments^93^. To ready bio-ISM technologies for implementation in mission architectures, scaling and adaptation of synthetic biology and bioprocess engineering to the relevant (off-world) environments (specifically Moon and Mars) is needed^16,94^. To this end, the appropriate hardware must be co-developed with the respective microbial cell factories for in-space bio-ISM^95^. The required investments and their allocation to realize this goal have recently been outlined^96^.

To evolve the technological readiness of space bio-ISM furthermore requires scientists and engineers from various fields, spanning biology, chemistry, physics, and engineering, to work together and build cross-compatible and scalable processing systems within the confines and stressors of space^16^. Biomolecular, bioprocess, and biosystems engineering must be integrated with pre-processing of resources and downstream processing of products, and tied in with mission-support infrastructure and logistics. Coordination mission specialists are critical to deploy tests in space under different (off-world) constraints and build long-term partnerships and understanding between the public and private sectors. Such groundwork requires long-lived multidisciplinary centers that are secure from volatility of markets and swings of political agendas to perform the large-scale, long-term science that is necessary to succeed. A dedicated space-based R&D Hub as an associated ‘field-station’ could greatly streamline and facilitate the advancement of fundamental technology that increases TRLs. Service providers would dedicate and manage resources both on the ISS (near-term), next-generation space station(s) (mid-term), and the Moon as test-bed for Mars (long-term). This pipeline would ensure testing, prototyping, and maturation of technologies in space with assigned, predictable launches, hardware and support.

### Prioritizing bio-ISM technology development

The strategic application of biomanufacturing has the potential to de-risk and expand crewed space-exploration capabilities. The farther from Earth the more mission-critical biomanufacturing becomes—Lunar missions may be not sustained only supplemented with LC, recycling and re-purposing of waste-streams, Mars missions will require ISRU. To take full advantage of mission supplies and in situ resources, advanced biomanufacturing technologies must be developed—given the austerity of the Moon and Mars, research efforts must be geared towards the most abundant resources to benefit future deep-space missions. Near-term Lunar missions will serve to build-out and stress-test LC technologies that will inform long-term ISM processes on Mars. Techno-economic assessment of mission-design scenarios directs the strategic development goals and can, as opposed to hardware, be readily implemented. Resource efficiency will be essential to successful deep-space missions, but it is also a criterion that is becoming increasingly salient amid our changing world. Developing technologies that operate with circularity at their core have significant potential to bring opportunities to imminent challenges on Earth.

## Supplementary information

Supplementary Information

Supplementary Dataset 1

Description of Additional Supplementary Files

## Data Availability

Supplementary materials include: (1) Supplementary Information document containing supporting and additional information such as a rendering of data used to produce Fig. 2; (2) Supplementary Dataset 1 including Jupyter notebook for plotting the results using spreadsheets as input. The provided materials are sufficient for reproducing all results, additional data can be requested from the corresponding authors.
