# Supplementary material for: Microbial biomanufacturing for space-exploration—what to take and when to make
Source: Nat Commun. 2023 Apr 21;14:2311. doi: 10.1038/s41467-023-37910-1 (PMC10121718; doi:10.1038/s41467-023-37910-1)
Supplement: Supplementary file 1 — Supplementary Information [file 41467_2023_37910_MOESM1_ESM.pdf]

Supplemental Information to: Microbial Biomanufacturing for Space Exploration – What to Take and When to Make

Nils J.H. Averesch[1,2,†,*], Aaron J. Berliner[1,3,†,*], Shannon N. Nangle[4,5,†,*], Spencer Zezulka[1,3,6], Gretchen L. Vengerova[1,3], Davian Ho[1,3], Cameran A. Casale[1,3], Benjamin A.E. Lehner[7], Jessica E. Snyder[8], Kevin B. Clark[9,10], Lewis R. Dartnell[11], Craig S. Criddle[1,2], and Adam P. Arkin[1,3]

[1]Center for the Utilization of Biological Engineering in Space (CUBES), USA
[2]Department of Civil and Environmental Engineering, Stanford University, Stanford, CA, USA
[3]Department of Bioengineering, University of California Berkeley, Berkeley, CA, USA
[4]Wyss Institute for Biologically Inspired Engineering at Harvard University, Boston, MA, USA
[5]Circe Bioscience Inc., Somerville, MA, USA
[6]School of Information, University of California Berkeley, Berkeley, CA, USA
[7]Department of Bionanoscience, Delft University of Technology, Delft, South Holland, NL
[8]Blue Marble Space Institute of Science, Seattle, WA, USA
[9]Cures Within Reach, Chicago, IL, USA
[10]Champions Program, eXtreme Science and Engineering Discovery Environment (XSEDE), USA
[11]Department of Life Sciences, University of Westminster, London, UK

[†]These authors contributed equally
[*]Corresponding Author Emails:
NJHA: nils.averesch@uq.net.au,
AJB: aaron.berliner@berkeley.edu,
SNN: shannon@circebioscience.com

1 Comparison of *In Situ* Manufacturing Approaches

Supplementary Table 1. Qualitative comparison of biotic vs. abiotic *in situ* (bio)manufacturing approaches for different off-Earth destinations (excluding *cis*-Lunar, which can be considered as an in-between of Earth Orbit and destination Moon).

**Destination**		**abiotic approach**	**biological approach**
**Earth Orbit**[1]	*advantage*	manufacturing of high-quality premium products	bioprinting without structural stabilization and scaffold-free tissue engineering
		re-use/-purposing of infrastructure on-orbit for strategic reduction of lift requirements	more extensive recycling of resources for better loop-closure and reduction of resupply
	drawback	no *in situ* resource utilization possible	no *in situ* resource utilization possible
		in many cases ability to re-supply outweighs infrastructure-investment	in many cases ability to re-supply outweighs infrastructure-investment
		microgravity makes certain processes more difficult	microgravity makes aqueous processes challenging
**Luna**[2]	*advantage*	strategic outpost for infrastructure as stepping stone to the solar system	exploitation of *in situ* resources that are not or hardly accessible otherwise
		partial gravity may allow certain processes to be adopted more readily	partial gravity allows gas/liquid separation, facilitating operation of aqueous processes
		may in some cases allow saving mass/-cost of re-supply	may in some cases allow expanded mission capabilities
	drawback	limited portfolio and low amount/density of available resources	resources are limited and processes are largely dependent on abiotic ISRU
		ability to re-supply and delivery may often outweigh infrastructure investment	ability to re-supply and delivery may sometimes outweigh infrastructure investment
**Interplanetary Transit**[3]	*advantage*	inability to re-supply demands recycling and loop-closure	increased redundancy through flexibility: *ad hoc* solution of complex incidental problems
		systems proven in LEO are readily translated/adapted	ability for more complete loop-closure and recycling
	drawback	no *in situ* resources available (only recycling/production from stock possible)	no *in situ* resources available (only recycling/production from stock possible)
		microgravity makes certain processes more difficult	microgravity makes aqueous processes challenging
**Mars**[4]	*advantage*	lack of robust supply-chain and impossible just-in-time response demands ISRU, LC, and ISM	especially suited to leverage all available *in situ* resources to expand autonomous capabilities
			exploitation of *in situ* resources that are not or hardly accessible otherwise
		partial gravity may allow certain processes to be adopted more readily	partial gravity allows gas/liquid separation, facilitating operation of aqueous processes
	drawback	high infrastructure investment (CapEx)	high maintenance requirement (OpEx)
		lack of resilience out-weighs greater robustness in long-term	more susceptible to performance-drift than abiotic solutions

2 Assumptions and Methodology of Inventory Analysis

In order to construct a set of comparable mission-design scenarios, we leveraged NASA's *Advanced Life Support Sizing Analysis Tool* (ALSSAT)[5,6] to compile the cargo inventory of environmental control and life support systems[7]. Breaking these down for each scenario by means of Equivalent Systems Mass (ESM)[8], the basis for a techno-economic analysis was established. In its current form[9], the total ESM $\mathfrak{M}$ is defined only for operations at a specific location as the sum over the set of all systems as:

$$\mathfrak{M} = L_{\text{eq}} \sum_{i=1}^{\mathscr{A}} [\underbrace{(M_i \cdot M_{\text{eq}}) + (V_i \cdot V_{\text{eq}}) + (P_i \cdot P_{\text{eq}}) + (C_i \cdot C_{\text{eq}})}_{\mathfrak{M}_{\text{NCT}}} + \underbrace{(CT_i \cdot D \cdot CT_{eq})}_{\mathfrak{M}_{\text{CT}}}] \tag{1}$$

for subsystem $i \in \mathscr{A}$ of the ESM excluding crew-time $\mathfrak{M}_{\text{NCT}}$ and the ESM including crew-time $\mathfrak{M}_{\text{CT}}$ where M_i, V_i, P_i, C_i are the initial mass [kg], volume [m^3], power requirement [kW_e], and cooling requirement [kg/kW_{th}], D is the duration of the mission segment [sol], T_i is the crew-time requirement based on an astronaut crew-member (CM) [CM-h/sol], M_{eq} is the stowage factor accounting for additional structural masses for a subsystem such as shelving [kg/kg], V_{eq} is the mass equivalency factor for ti he pressurized volume support infrastructure [kg/m^3], P_{eq} is the mass equivalency factor for the power generation support infrastructure [kg/kW_e], C_{eq} is the mass equivalency factor for the cooling infrastructure [kg/kW_{th}], CT_{eq} is the mass equivalency factor for the crew-time [kg/CM-h], and L_{eq} is the location factor for the mission segment [kg/kg] which accounts for the cost to transport mass from one location in space to another (such as Earth-orbit to Mars-orbit). Mass equivalency factors (V_{eq}, P_{eq}, C_{eq}, CT_{eq}) are used to convert the non-mass parameters into a mass-unit[10].

2.1 Inventory Analysis by Equivalent Systems Mass

Scenario definitions were constructed and parameters were sourced from projections and values described in literature, primarily NASA's *Life Support Baseline Values and Assumptions Documentation* (BVAD)[11], as well as the ALSSAT[6] itself. The scenarios and associated parameters are outlined in Supplementary Table 2, which includes the scenario identifier (I to V), duration of surface mission operations in days, primary surface operation destination (Moon or Mars), and sortie number S_{num}. The sortie number corresponds to the number of "trips" for a given scenario. Also included are the equivalency factors (M_{eq}, V_{eq}, P_{eq}, C_{eq}, CT_{eq}, and L_{eq}) used to determine the ESM as per Eq. 1, allowing for the comparable calculation $\mathfrak{M}$.

Table Supplementary 2. Parameter description of exemplar scenarios—scenarios 'I' and 'II' correspond to single sorties ($N = 1$) to Moon and Mars respectively using standard surface operation duration[11], while scenarios 'III' and 'IV' correspond to multi-sortie campaigns with the same total surface operation as the single-sortie scenario 'V'. All scenarios consider a crew-strength of four astronauts. These parameters can be used to calculate the expense in form of ESM and include equivalency factors for volume (V_{eq}), power (P_{eq}), cooling (C_{eq}), crew-time (CT_{eq}), and location (L_{eq}).

Scenario	Duration	Destination	S_{num}	V_{eq}	P_{eq}	C_{eq}	CT_{eq}	L_{eq}
I	180	Moon	1	126	136	55.4	1.5	7.2
II	540	Mars	1	117.7	162	96	0.47	30
III	180	Moon	30	126	136	55.4	1.5	7.2
IV	540	Mars	10	117.7	162	96	0.47	30
V	5,400	Mars	1	117.7	162	96	0.47	30

The ALSSAT was then used to generate an exemplar set of inventory items for all systems and subsystems ($i \in \mathscr{A}$) as shown in Supplementary Table 3. The data-set in Supplementary Table 3 includes a uniformized breakdown for all inventory items by system, subsystem, and item name – as well as the ESM terms (M, V, P, C, CT) for each item in each scenario. Using the data from Supplementary Table 2 and Supplementary Table 3, the total ESM $\mathfrak{M}_t$ for each scenario was calculated using the form $\mathfrak{M}_t = S_{\text{num}} \mathfrak{M}_{S_{\text{num}}=1}$. The results are visualized in Supplementary Figure 1 and Supplementary Figure 2.

Acquisition of Equivalency and Location Factors

Standard mass equivalency factors can be found in the BVAD[11] as well as the ALSSAT[6]. The volume equivalency factor $V_{\mathbf{eq}}$ is the mass equivalency factor for the pressurized volume support infrastructure of subsystem i [kg/m^3]. For Lunar operations, this term was selected using an inflatable module from the BVAD[11] (Table 3-11). For Martian operations, this term was taken from the ALSSAT standard systems[6]. The power equivalency factor $P_{\mathbf{eq}}$ is the mass equivalency factor for the power generation support infrastructure of subsystem i in [kg/kW]. For both, Lunar and Martian operations, the terms were selected from the BVAD[11] (Table 3-16 and Table 3-5, respectively). The cooling/thermal equivalency factor $C_{\mathbf{eq}}$ is the the mass equivalency factor for the cooling infrastructure of subsystem i in [kg/kW]. For Lunar operations, this term was selected from the BVAD[11] (Table 3-3 and Table 3-19). For Martian operations, this term was taken from the ALSSAT standard systems[6]. The operation and maintenance of life-support equipment demands crewtime, which varies depending on the system. In particular, tasks related to food such as production, preparation and waste disposal can require a significant commitment of crewtime. The associated expenses are calculated based on the equivalent system mass (ESM) of the life-support system and the available crewtime. On average, the equivalent system mass ranges from 0.1 to 10 crew-member hours per kilogram of ESM. For both Luna and Mars, the crewtime equivalency factor $CT_{\mathbf{eq}}$ in [kg/h] was derived from the BVAD[11]. Additional considerations on adaptation of the crewtime equivalency factor can be found in previous research[10]. Location factors L_{eq} in [kg/kg] describes the extra resources required to transfer a payload from low-Earth orbit to another off-Earth location These resources include propulsion elements such as engines, tankage, fuel and associated propulsion structure. Location factors allow for comparison of scenarios where payloads do not have the same transportation history. For instance, one payload might remain on a single vehicle throughout the mission, while another might jettison mass during the mission, reducing its propulsion costs. The BVAD[11] contains the location factors for two destinations - Moon and Mars. For the Moon estimates are based on the L1 Gateway architecture while for Mars estimates are based on the Mars Dual Lander architecture, which conform to the Design Reference Mission Architecture[12]. Both sets of estimates assume the use of chemical propulsion and aero-braking when feasible.

2.2 Inventory Analysis by Elemental Composition

The inventory item composition analysis was carried out by first creating a set of composition classes, which were: Structural Metal, Plastic, Electronics, Fabric, Glass, Rubber, Ceramics, Gas, Biomass, Water, Other. These classes were created to allow *prima facie* estimates for the composition of individual items. Next, we estimated the fractional composition for each inventory item as shown in Supplementary Table 4. We note that these are approximations, based on a number of factors and derived from relevant sources such as NASA documentation, as well as various other forms of literature, product catalogs, etc. Despite our best effort, often assumptions had to be made in order to estimate the composition of individual items, which cannot be backed with precise data, simply because they do not exist. We argue that this is acceptable as individual accuracy is neither the goal of nor required for these preliminary calculations. Rather, this analysis should be considered an initial step towards defining the order of magnitudes for the composition of mission inventories.

Supplementary Table 3. ESM parameters for inventory items broken down by system. Parameters are mass M [kg], volume V [m^3], power P [kW], cooling C [kW], crew-time CT [h][11,13]. ESM values correspond to a single sortie $S_{num} = 1$ for the scenarios described in Supplementary Table 2. (ORU = Orbital Replacement Unit)

			A					B					C					D					E				
**System**	**Subsystem**	**Item**	**M**	**V**	**P**	**C**	**CT**	**M**	**V**	**P**	**C**	**CT**	**M**	**V**	**P**	**C**	**CT**	**M**	**V**	**P**	**C**	**CT**	**M**	**V**	**P**	**C**	**CT**
Air	APC	Vent/Relief Valve	5.40	0.01	0.00	0.00	0.00	5.40	0.01	0.00	0.00	0.00	5.40	0.01	0.00	0.00	0.00	5.40	0.01	0.00	0.00	0.00	5.40	0.01	0.00	0.00	0.00
Air	APC	Pressure Control Panel	11.20	0.03	18.00	18.00	0.00	11.20	0.03	18.00	18.00	0.00	11.20	0.03	18.00	18.00	0.00	11.20	0.03	18.00	18.00	0.00	11.20	0.03	18.00	18.00	0.00
Air	APC	Manual Pressure Equalization Valve	9.60	0.01	0.00	0.00	0.00	9.60	0.01	0.00	0.00	0.00	9.60	0.01	0.00	0.00	0.00	9.60	0.01	0.00	0.00	0.00	9.60	0.01	0.00	0.00	0.00
Air	APC	Positive Pressure Relief Valve	1.80	0.00	0.00	0.00	0.00	1.80	0.00	0.00	0.00	0.00	1.80	0.00	0.00	0.00	0.00	1.80	0.00	0.00	0.00	0.00	1.80	0.00	0.00	0.00	0.00
Air	APC	Negative Pressure Relief Valve	3.00	0.01	0.00	0.00	0.00	3.00	0.01	0.00	0.00	0.00	3.00	0.01	0.00	0.00	0.00	3.00	0.01	0.00	0.00	0.00	3.00	0.01	0.00	0.00	0.00
Air	APC	Nitrogen Interface Assembly	7.50	0.01	5.50	5.50	0.00	7.50	0.01	5.50	5.50	0.00	7.50	0.01	5.50	5.50	0.00	7.50	0.01	5.50	5.50	0.00	7.50	0.01	5.50	5.50	0.00
Air	APC	Vacuum Access Jumper 5-ft	0.70	0.00	0.00	0.00	0.00	0.70	0.00	0.00	0.00	0.00	0.70	0.00	0.00	0.00	0.00	0.70	0.00	0.00	0.00	0.00	0.70	0.00	0.00	0.00	0.00
Air	APC	Vacuum Access Jumper 35-ft	3.20	0.00	0.00	0.00	0.00	3.20	0.00	0.00	0.00	0.00	3.20	0.00	0.00	0.00	0.00	3.20	0.00	0.00	0.00	0.00	3.20	0.00	0.00	0.00	0.00
Air	ACMA	Verification Gas Assembly	5.40	0.01	0.10	0.10	0.00	5.40	0.01	0.10	0.10	0.00	5.40	0.01	0.10	0.10	0.00	5.40	0.01	0.10	0.10	0.00	5.40	0.01	0.10	0.10	0.00
Air	ACMA	Mass Spectrometer	13.90	0.02	31.80	31.80	0.00	13.90	0.02	31.80	31.80	0.00	13.90	0.02	31.80	31.80	0.00	13.90	0.02	31.80	31.80	0.00	13.90	0.02	31.80	31.80	0.00
Air	ACMA	Sample Pump	3.40	0.00	4.00	4.00	0.00	3.40	0.00	4.00	4.00	0.00	3.40	0.00	4.00	4.00	0.00	3.40	0.00	4.00	4.00	0.00	3.40	0.00	4.00	4.00	0.00
Air	ACMA	Sample Distributor	2.10	0.00	0.10	0.10	0.00	2.10	0.00	0.10	0.10	0.00	2.10	0.00	0.10	0.10	0.00	2.10	0.00	0.10	0.10	0.00	2.10	0.00	0.10	0.10	0.00
Air	ACMA	Data + Control	8.00	0.01	34.90	34.90	0.00	8.00	0.01	34.90	34.90	0.00	8.00	0.01	34.90	34.90	0.00	8.00	0.01	34.90	34.90	0.00	8.00	0.01	34.90	34.90	0.00
Air	ACMA	Low Voltage Power Supply	5.70	0.01	30.80	30.80	0.00	5.70	0.01	30.80	30.80	0.00	5.70	0.01	30.80	30.80	0.00	5.70	0.01	30.80	30.80	0.00	5.70	0.01	30.80	30.80	0.00
Air	ACMA	Chassis	15.80	0.02	0.00	0.00	0.00	15.80	0.02	0.00	0.00	0.00	15.80	0.02	0.00	0.00	0.00	15.80	0.02	0.00	0.00	0.00	15.80	0.02	0.00	0.00	0.00
Air	ACMA	Inlet Valve Assembly	0.00	0.00	0.00	0.00	0.00	0.00	0.00	0.00	0.00	0.00	0.00	0.00	0.00	0.00	0.00	0.00	0.00	0.00	0.00	0.00	0.00	0.00	0.00	0.00	0.00
Air	ACMA	EMI Filter	0.00	0.00	1.80	1.80	0.00	0.00	0.00	1.80	1.80	0.00	0.00	0.00	1.80	1.80	0.00	0.00	0.00	1.80	1.80	0.00	0.00	0.00	1.80	1.80	0.00
Air	SDS	3-way Solenoid Valves	31.50	0.03	0.00	0.00	0.00	31.50	0.03	0.00	0.00	0.00	31.50	0.03	0.00	0.00	0.00	31.50	0.03	0.00	0.00	0.00	31.50	0.03	0.00	0.00	0.00
Air	SDS	Manual Valves	2.53	0.01	0.00	0.00	0.00	2.53	0.01	0.00	0.00	0.00	2.53	0.01	0.00	0.00	0.00	2.53	0.01	0.00	0.00	0.00	2.53	0.01	0.00	0.00	0.00
Air	SDS	Sample probes	1.08	0.00	0.00	0.00	0.00	1.08	0.00	0.00	0.00	0.00	1.08	0.00	0.00	0.00	0.00	1.08	0.00	0.00	0.00	0.00	1.08	0.00	0.00	0.00	0.00
Air	CO_2	Air Selector Valve	12.81	0.01	0.70	0.70	0.99	12.81	0.01	0.70	0.70	0.99	12.75	0.01	0.70	0.70	0.99	12.65	0.01	0.68	0.68	2.74	12.60	0.01	0.68	0.68	29.59
Air	CO_2	Desiccant Bed	24.29	0.25	0.00	0.00	0.24	24.29	0.25	0.00	0.00	0.24	24.07	0.25	0.00	0.00	0.24	23.69	0.24	0.00	0.00	0.66	23.50	0.24	0.00	0.00	7.10
Air	CO_2	Adsorbent Bed	33.13	0.00	578.43	578.43	0.00	33.13	0.00	578.43	578.43	0.00	32.83	0.00	573.16	573.16	0.00	32.32	0.00	564.28	564.28	0.00	32.06	0.00	559.74	559.74	0.00
Air	CO_2	Air Check Valve	0.16	0.00	0.00	0.00	0.00	0.16	0.00	0.00	0.00	0.00	0.16	0.00	0.00	0.00	0.00	0.16	0.00	0.00	0.00	0.00	0.16	0.00	0.00	0.00	0.00
Air	CO_2	Heater Controller	6.60	0.00	38.00	38.00	0.01	6.60	0.00	38.00	38.00	0.01	6.60	0.00	38.00	38.00	0.01	6.60	0.00	38.00	38.00	0.03	6.60	0.00	38.00	38.00	0.30
Air	CO_2	Air Blower	0.82	0.02	40.71	40.71	0.06	0.82	0.02	40.71	40.71	0.06	0.82	0.02	40.34	40.34	0.06	0.81	0.02	39.72	39.72	0.18	0.81	0.02	39.40	39.40	1.92
Air	CO_2	Pre-cooler	2.22	0.00	0.00	0.00	0.00	2.22	0.00	0.00	0.00	0.00	2.21	0.00	0.00	0.00	0.00	2.19	0.00	0.00	0.00	0.00	2.18	0.00	0.00	0.00	0.00
Air	CO_2	Blower/Pre-cooler Motor Controller	1.30	0.00	5.00	5.00	0.00	1.30	0.00	5.00	5.00	0.00	1.30	0.00	5.00	5.00	0.00	1.30	0.00	5.00	5.00	0.00	1.30	0.00	5.00	5.00	0.00
Air	CO_2	CO_2 Pump	6.73	0.00	13.34	13.34	0.04	6.73	0.00	13.34	13.34	0.04	6.70	0.00	13.22	13.22	0.04	6.65	0.00	13.01	13.01	0.11	6.62	0.00	12.91	12.91	1.18
Air	CO_2	CO_2 Pump Motor Controller	1.30	0.01	2.00	2.00	0.00	1.30	0.01	2.00	2.00	0.00	1.30	0.01	2.00	2.00	0.00	1.30	0.01	2.00	2.00	0.01	1.30	0.01	2.00	2.00	0.15
Air	CO_2	Temperature Sensor	0.40	0.00	1.00	1.00	0.00	0.40	0.00	1.00	1.00	0.00	0.40	0.00	1.00	1.00	0.00	0.40	0.00	1.00	1.00	0.00	0.40	0.00	1.00	1.00	0.00
Air	CO_2	Differential Pressure Sensor	0.20	0.00	1.00	1.00	0.00	0.20	0.00	1.00	1.00	0.00	0.20	0.00	1.00	1.00	0.00	0.20	0.00	1.00	1.00	0.00	0.20	0.00	1.00	1.00	0.00
Air	CO_2	Absolute Pressure Sensor	0.20	0.00	1.00	1.00	0.00	0.20	0.00	1.00	1.00	0.00	0.20	0.00	1.00	1.00	0.00	0.20	0.00	1.00	1.00	0.00	0.20	0.00	1.00	1.00	0.00
Air	CO_2	Electrical Harness	4.50	0.00	0.00	0.00	0.00	4.50	0.00	0.00	0.00	0.00	4.50	0.00	0.00	0.00	0.00	4.50	0.00	0.00	0.00	0.00	4.50	0.00	0.00	0.00	0.00
Air	CO_2	Plumbing	4.85	0.00	0.00	0.00	0.00	4.85	0.00	0.00	0.00	0.00	4.82	0.00	0.00	0.00	0.00	4.78	0.00	0.00	0.00	0.00	4.76	0.00	0.00	0.00	0.00
Air	CO_2	Support Structure	29.64	0.00	0.00	0.00	0.00	29.64	0.00	0.00	0.00	0.00	29.51	0.00	0.00	0.00	0.00	29.27	0.00	0.00	0.00	0.00	29.15	0.00	0.00	0.00	0.00
Air	CO_2	Fluid Disconnects	1.97	0.00	0.00	0.00	0.00	1.97	0.00	0.00	0.00	0.00	1.96	0.00	0.00	0.00	0.00	1.95	0.00	0.00	0.00	0.00	1.94	0.00	0.00	0.00	0.00

Supplementary Table 3. ESM parameters for inventory items broken down by system. Parameters are mass M [kg], volume V [m^3], power P [kW], cooling C [kW], crew-time CT [h][11,13]. ESM values correspond to a single sortie $S_{\mathrm{num}} = 1$ for the scenarios described in Supplementary Table 2. (ORU = Orbital Replacement Unit)

			A					B					C					D					E				
**System**	**Subsystem**	**Item**	**M**	**V**	**P**	**C**	**CT**	**M**	**V**	**P**	**C**	**CT**	**M**	**V**	**P**	**C**	**CT**	**M**	**V**	**P**	**C**	**CT**	**M**	**V**	**P**	**C**	**CT**
Air	CO_2	Electronics Cold-Plate	2.71	0.00	0.00	0.00	0.00	2.71	0.00	0.00	0.00	0.00	2.70	0.00	0.00	0.00	0.00	2.68	0.00	0.00	0.00	0.00	2.67	0.00	0.00	0.00	0.00
Air	CO_2	Electronics Interface Plate	1.60	0.00	0.00	0.00	0.00	1.60	0.00	0.00	0.00	0.00	1.60	0.00	0.00	0.00	0.00	1.60	0.00	0.00	0.00	0.00	1.60	0.00	0.00	0.00	0.00
Air	N_2	MD Shield Instl	0.00	0.00	0.00	0.00	0.00	0.00	0.00	0.00	0.00	0.00	0.00	0.00	0.00	0.00	0.00	0.00	0.00	0.00	0.00	0.00	0.00	0.00	0.00	0.00	0.00
Air	N_2	Multilayer Insulation Assembly-T #1	0.00	0.00	0.00	0.00	0.00	0.00	0.00	0.00	0.00	0.00	0.00	0.00	0.00	0.00	0.00	0.00	0.00	0.00	0.00	0.00	0.00	0.00	0.00	0.00	0.00
Air	N_2	Multilayer Insulation Assembly-T #2	0.00	0.00	0.00	0.00	0.00	0.00	0.00	0.00	0.00	0.00	0.00	0.00	0.00	0.00	0.00	0.00	0.00	0.00	0.00	0.00	0.00	0.00	0.00	0.00	0.00
Air	N_2	Primary Structure Assembly-HPG ORU	0.00	0.00	0.00	0.00	0.00	0.00	0.00	0.00	0.00	0.00	0.00	0.00	0.00	0.00	0.00	0.00	0.00	0.00	0.00	0.00	0.00	0.00	0.00	0.00	0.00
Air	N_2	Tank ORU Assembly	0.00	0.00	0.00	0.00	0.00	0.00	0.00	0.00	0.00	0.00	0.00	0.00	0.00	0.00	0.00	0.00	0.00	0.00	0.00	0.00	0.00	0.00	0.00	0.00	0.00
Air	N_2	Utilities Installation - O_2/N_2 Tank	0.00	0.00	0.00	0.00	0.00	0.00	0.00	0.00	0.00	0.00	0.00	0.00	0.00	0.00	0.00	0.00	0.00	0.00	0.00	0.00	0.00	0.00	0.00	0.00	0.00
Air	N_2	N_2 Bare Tank	80.37	0.00	0.00	0.00	0.00	80.37	0.00	0.00	0.00	0.00	80.43	0.00	0.00	0.00	0.00	163.98	0.00	0.00	0.00	0.00	1443.26	0.00	0.00	0.00	0.00
Air	N_2	HPGA Fluid	81.74	0.00	0.00	0.00	0.00	81.74	0.00	0.00	0.00	0.00	81.83	0.00	0.00	0.00	0.00	144.73	0.00	0.00	0.00	0.00	1107.91	0.00	0.00	0.00	0.00
Air	N_2	Handhold, top mounted	0.22	0.00	0.00	0.00	0.00	0.22	0.00	0.00	0.00	0.00	0.22	0.00	0.00	0.00	0.00	0.38	0.00	0.00	0.00	0.00	2.92	0.00	0.00	0.00	0.00
Air	N_2	Handrail 21.941 in custom	0.38	0.00	0.00	0.00	0.00	0.38	0.00	0.00	0.00	0.00	0.38	0.00	0.00	0.00	0.00	0.67	0.00	0.00	0.00	0.00	5.12	0.00	0.00	0.00	0.00
Air	N_2	Handrail, top mounted	0.40	0.00	0.00	0.00	0.00	0.40	0.00	0.00	0.00	0.00	0.40	0.00	0.00	0.00	0.00	0.70	0.00	0.00	0.00	0.00	5.36	0.00	0.00	0.00	0.00
Air	N_2	Grapple Fixture, flt releasable	11.11	0.00	0.00	0.00	0.00	11.11	0.00	0.00	0.00	0.00	11.13	0.00	0.00	0.00	0.00	19.68	0.00	0.00	0.00	0.00	150.63	0.00	0.00	0.00	0.00
Air	N_2	Accessories	4.02	0.00	0.00	0.00	0.00	4.02	0.00	0.00	0.00	0.00	4.03	0.00	0.00	0.00	0.00	8.21	0.00	0.00	0.00	0.00	72.24	0.00	0.00	0.00	0.00
Air	O_2	MD Shield Instl	0.00	0.00	0.00	0.00	0.00	0.00	0.00	0.00	0.00	0.00	0.00	0.00	0.00	0.00	0.00	0.00	0.00	0.00	0.00	0.00	0.00	0.00	0.00	0.00	0.00
Air	O_2	Multilayer Insulation Assembly-T #1	0.00	0.00	0.00	0.00	0.00	0.00	0.00	0.00	0.00	0.00	0.00	0.00	0.00	0.00	0.00	0.00	0.00	0.00	0.00	0.00	0.00	0.00	0.00	0.00	0.00
Air	O_2	Multilayer Insulation Assembly-T #2	0.00	0.00	0.00	0.00	0.00	0.00	0.00	0.00	0.00	0.00	0.00	0.00	0.00	0.00	0.00	0.00	0.00	0.00	0.00	0.00	0.00	0.00	0.00	0.00	0.00
Air	O_2	Primary Structure Assembly-HPG ORU	0.00	0.00	0.00	0.00	0.00	0.00	0.00	0.00	0.00	0.00	0.00	0.00	0.00	0.00	0.00	0.00	0.00	0.00	0.00	0.00	0.00	0.00	0.00	0.00	0.00
Air	O_2	Tank ORU Assembly	0.00	0.00	0.00	0.00	0.00	0.00	0.00	0.00	0.00	0.00	0.00	0.00	0.00	0.00	0.00	0.00	0.00	0.00	0.00	0.00	0.00	0.00	0.00	0.00	0.00
Air	O_2	Utilities Installation - O_2/N_2 Tank	0.00	0.00	0.00	0.00	0.00	0.00	0.00	0.00	0.00	0.00	0.00	0.00	0.00	0.00	0.00	0.00	0.00	0.00	0.00	0.00	0.00	0.00	0.00	0.00	0.00
Air	O_2	O_2 Bare Tank	56.08	0.00	0.00	0.00	0.00	56.08	0.00	0.00	0.00	0.00	53.53	0.00	0.00	0.00	0.00	112.19	0.00	0.00	0.00	0.00	1010.35	0.00	0.00	0.00	0.00
Air	O_2	HPGA Fluid	48.00	0.00	0.00	0.00	0.00	48.00	0.00	0.00	0.00	0.00	44.47	0.00	0.00	0.00	0.00	72.80	0.00	0.00	0.00	0.00	506.64	0.00	0.00	0.00	0.00
Air	O_2	Handhold, top mounted	0.13	0.00	0.00	0.00	0.00	0.13	0.00	0.00	0.00	0.00	0.12	0.00	0.00	0.00	0.00	0.19	0.00	0.00	0.00	0.00	1.34	0.00	0.00	0.00	0.00
Air	O_2	Handrail 21.941 in custom	0.22	0.00	0.00	0.00	0.00	0.22	0.00	0.00	0.00	0.00	0.21	0.00	0.00	0.00	0.00	0.34	0.00	0.00	0.00	0.00	2.34	0.00	0.00	0.00	0.00
Air	O_2	Handrail, top mounted	0.23	0.00	0.00	0.00	0.00	0.23	0.00	0.00	0.00	0.00	0.22	0.00	0.00	0.00	0.00	0.35	0.00	0.00	0.00	0.00	2.45	0.00	0.00	0.00	0.00
Air	O_2	Grapple Fixture, flt releasable	6.53	0.00	0.00	0.00	0.00	6.53	0.00	0.00	0.00	0.00	6.05	0.00	0.00	0.00	0.00	9.90	0.00	0.00	0.00	0.00	68.88	0.00	0.00	0.00	0.00
Air	O_2	Accessories	2.81	0.00	0.00	0.00	0.00	2.81	0.00	0.00	0.00	0.00	2.68	0.00	0.00	0.00	0.00	5.62	0.00	0.00	0.00	0.00	50.57	0.00	0.00	0.00	0.00
Air	Sabatier	Condensing Heat Exchanger	1.47	0.00	0.00	0.00	0.00	1.47	0.00	0.00	0.00	0.00	1.49	0.00	0.00	0.00	0.00	1.48	0.00	0.00	0.00	0.00	1.48	0.00	0.00	0.00	0.00
Air	Sabatier	AAA Heat Exchanger	2.47	0.00	0.00	0.00	0.00	2.47	0.00	0.00	0.00	0.00	2.50	0.00	0.00	0.00	0.00	2.49	0.00	0.00	0.00	0.00	2.49	0.00	0.00	0.00	0.00
Air	Sabatier	ITCS Coolant Water Inlet QD	0.47	0.00	0.00	0.00	0.00	0.47	0.00	0.00	0.00	0.00	0.48	0.00	0.00	0.00	0.00	0.48	0.00	0.00	0.00	0.00	0.48	0.00	0.00	0.00	0.00
Air	Sabatier	ITCS Coolant Water Outlet QD	0.36	0.00	0.00	0.00	0.00	0.36	0.00	0.00	0.00	0.00	0.36	0.00	0.00	0.00	0.00	0.36	0.00	0.00	0.00	0.00	0.36	0.00	0.00	0.00	0.00
Air	Sabatier	Heat Exchanger Inlet Temp	0.36	0.00	0.00	0.00	0.00	0.36	0.00	0.00	0.00	0.00	0.36	0.00	0.00	0.00	0.00	0.36	0.00	0.00	0.00	0.00	0.36	0.00	0.00	0.00	0.00
Air	Sabatier	Heat Exchanger Outlet Temp	0.36	0.00	0.00	0.00	0.00	0.36	0.00	0.00	0.00	0.00	0.36	0.00	0.00	0.00	0.00	0.36	0.00	0.00	0.00	0.00	0.36	0.00	0.00	0.00	0.00
Air	Sabatier	Manifold, CO_2	4.72	0.00	0.00	0.00	0.00	4.72	0.00	0.00	0.00	0.00	4.80	0.00	0.00	0.00	0.00	4.77	0.00	0.00	0.00	0.00	4.76	0.00	0.00	0.00	0.00
Air	Sabatier	CO_2 Inlet Check Valve	0.10	0.00	0.00	0.00	0.00	0.10	0.00	0.00	0.00	0.00	0.11	0.00	0.00	0.00	0.00	0.11	0.00	0.00	0.00	0.00	0.11	0.00	0.00	0.00	0.00
Air	Sabatier	CO_2 Inlet Filter	0.05	0.00	0.00	0.00	0.00	0.05	0.00	0.00	0.00	0.00	0.06	0.00	0.00	0.00	0.00	0.06	0.00	0.00	0.00	0.00	0.06	0.00	0.00	0.00	0.00

Supplementary Table 3. ESM parameters for inventory items broken down by system. Parameters are mass M [kg], volume V [m^3], power P [kW], cooling C [kW], crew-time CT [h][11,13]. ESM values correspond to a single sortie $S_{\text{num}} = 1$ for the scenarios described in Supplementary Table 2. (ORU = Orbital Replacement Unit)

			A					B					C					D					E				
**System**	**Subsystem**	**Item**	**M**	**V**	**P**	**C**	**CT**	**M**	**V**	**P**	**C**	**CT**	**M**	**V**	**P**	**C**	**CT**	**M**	**V**	**P**	**C**	**CT**	**M**	**V**	**P**	**C**	**CT**
Air	Sabatier	Pressure Sensor, CO_2 Inlet	0.41	0.00	0.00	0.00	0.00	0.41	0.00	0.00	0.00	0.00	0.41	0.00	0.00	0.00	0.00	0.41	0.00	0.00	0.00	0.00	0.41	0.00	0.00	0.00	0.00
Air	Sabatier	CO_2 Inlet QD	0.47	0.00	0.00	0.00	0.00	0.47	0.00	0.00	0.00	0.00	0.48	0.00	0.00	0.00	0.00	0.48	0.00	0.00	0.00	0.00	0.48	0.00	0.00	0.00	0.00
Air	Sabatier	CO_2 Inlet Regulator	0.92	0.00	0.00	0.00	0.00	0.92	0.00	0.00	0.00	0.00	0.93	0.00	0.00	0.00	0.00	0.93	0.00	0.00	0.00	0.00	0.93	0.00	0.00	0.00	0.00
Air	Sabatier	CO_2 Inlet NC Solenoid	0.47	0.00	0.00	0.00	0.00	0.47	0.00	0.00	0.00	0.00	0.48	0.00	0.00	0.00	0.00	0.48	0.00	0.00	0.00	0.00	0.48	0.00	0.00	0.00	0.00
Air	Sabatier	CO_2 Inlet Flow Control	2.15	0.00	0.00	0.00	0.00	2.15	0.00	0.00	0.00	0.00	2.18	0.00	0.00	0.00	0.00	2.17	0.00	0.00	0.00	0.00	2.17	0.00	0.00	0.00	0.00
Air	Sabatier	CO_2 Flow Control Orifice	0.05	0.00	0.00	0.00	0.00	0.05	0.00	0.00	0.00	0.00	0.05	0.00	0.00	0.00	0.00	0.05	0.00	0.00	0.00	0.00	0.05	0.00	0.00	0.00	0.00
Air	Sabatier	Delta P Sensor, Flow Sensor CO_2	0.91	0.00	0.00	0.00	0.00	0.91	0.00	0.00	0.00	0.00	0.91	0.00	0.00	0.00	0.00	0.91	0.00	0.00	0.00	0.00	0.91	0.00	0.00	0.00	0.00
Air	Sabatier	CO_2 Flow Meter Orifice	0.03	0.00	0.00	0.00	0.00	0.03	0.00	0.00	0.00	0.00	0.03	0.00	0.00	0.00	0.00	0.03	0.00	0.00	0.00	0.00	0.03	0.00	0.00	0.00	0.00
Air	Sabatier	Manifold, Hydrogen	4.38	0.00	0.00	0.00	0.00	4.38	0.00	0.00	0.00	0.00	4.45	0.00	0.00	0.00	0.00	4.43	0.00	0.00	0.00	0.00	4.42	0.00	0.00	0.00	0.00
Air	Sabatier	Water Outlet Quick Disconnect	0.47	0.00	0.00	0.00	0.00	0.47	0.00	0.00	0.00	0.00	0.48	0.00	0.00	0.00	0.00	0.48	0.00	0.00	0.00	0.00	0.48	0.00	0.00	0.00	0.00
Air	Sabatier	Hydrogen Inlet Check Valve	0.10	0.00	0.00	0.00	0.00	0.10	0.00	0.00	0.00	0.00	0.11	0.00	0.00	0.00	0.00	0.11	0.00	0.00	0.00	0.00	0.11	0.00	0.00	0.00	0.00
Air	Sabatier	Hydrogen Inlet Filter	0.05	0.00	0.00	0.00	0.00	0.05	0.00	0.00	0.00	0.00	0.06	0.00	0.00	0.00	0.00	0.06	0.00	0.00	0.00	0.00	0.06	0.00	0.00	0.00	0.00
Air	Sabatier	H_2O Outlet Pressure Sensor	0.82	0.00	0.00	0.00	0.00	0.82	0.00	0.00	0.00	0.00	0.82	0.00	0.00	0.00	0.00	0.82	0.00	0.00	0.00	0.00	0.82	0.00	0.00	0.00	0.00
Air	Sabatier	Hydrogen Inlet Quick Disconnect	0.47	0.00	0.00	0.00	0.00	0.47	0.00	0.00	0.00	0.00	0.48	0.00	0.00	0.00	0.00	0.48	0.00	0.00	0.00	0.00	0.48	0.00	0.00	0.00	0.00
Air	Sabatier	Hydrogen Inlet NC Solenoid	0.94	0.00	0.00	0.00	0.00	0.94	0.00	0.00	0.00	0.00	0.96	0.00	0.00	0.00	0.00	0.95	0.00	0.00	0.00	0.00	0.95	0.00	0.00	0.00	0.00
Air	Sabatier	Delta P Sensor, Flow Sensor H_2	0.91	0.00	0.00	0.00	0.00	0.91	0.00	0.00	0.00	0.00	0.91	0.00	0.00	0.00	0.00	0.91	0.00	0.00	0.00	0.00	0.91	0.00	0.00	0.00	0.00
Air	Sabatier	H_2 Flow Meter Orifice	0.03	0.00	0.00	0.00	0.00	0.03	0.00	0.00	0.00	0.00	0.03	0.00	0.00	0.00	0.00	0.03	0.00	0.00	0.00	0.00	0.03	0.00	0.00	0.00	0.00
Air	Sabatier	Manifold, Vent	5.06	0.00	0.00	0.00	0.00	5.06	0.00	0.00	0.00	0.00	5.14	0.00	0.00	0.00	0.00	5.12	0.00	0.00	0.00	0.00	5.10	0.00	0.00	0.00	0.00
Air	Sabatier	Liquid Sensor	1.09	0.00	0.00	0.00	0.00	1.09	0.00	0.00	0.00	0.00	1.09	0.00	0.00	0.00	0.00	1.09	0.00	0.00	0.00	0.00	1.09	0.00	0.00	0.00	0.00
Air	Sabatier	Vent Pressure Sensor	0.82	0.00	0.00	0.00	0.00	0.82	0.00	0.00	0.00	0.00	0.82	0.00	0.00	0.00	0.00	0.82	0.00	0.00	0.00	0.00	0.82	0.00	0.00	0.00	0.00
Air	Sabatier	Vent Outlet Quick Disconnect	0.47	0.00	0.00	0.00	0.00	0.47	0.00	0.00	0.00	0.00	0.48	0.00	0.00	0.00	0.00	0.48	0.00	0.00	0.00	0.00	0.48	0.00	0.00	0.00	0.00
Air	Sabatier	Vent Regulator	0.92	0.00	0.00	0.00	0.00	0.92	0.00	0.00	0.00	0.00	0.93	0.00	0.00	0.00	0.00	0.93	0.00	0.00	0.00	0.00	0.93	0.00	0.00	0.00	0.00
Air	Sabatier	Vent Relief/Check #1	0.16	0.00	0.00	0.00	0.00	0.16	0.00	0.00	0.00	0.00	0.16	0.00	0.00	0.00	0.00	0.16	0.00	0.00	0.00	0.00	0.16	0.00	0.00	0.00	0.00
Air	Sabatier	Vent Relief/Check #2	0.16	0.00	0.00	0.00	0.00	0.16	0.00	0.00	0.00	0.00	0.16	0.00	0.00	0.00	0.00	0.16	0.00	0.00	0.00	0.00	0.16	0.00	0.00	0.00	0.00
Air	Sabatier	Vent Outlet NO Solenoid	0.94	0.00	0.00	0.00	0.00	0.94	0.00	0.00	0.00	0.00	0.96	0.00	0.00	0.00	0.00	0.95	0.00	0.00	0.00	0.00	0.95	0.00	0.00	0.00	0.00
Air	Sabatier	Water Pressure Sensor	0.82	0.00	0.00	0.00	0.00	0.82	0.00	0.00	0.00	0.00	0.82	0.00	0.00	0.00	0.00	0.82	0.00	0.00	0.00	0.00	0.82	0.00	0.00	0.00	0.00
Air	Sabatier	Water Relief	0.16	0.00	0.00	0.00	0.00	0.16	0.00	0.00	0.00	0.00	0.16	0.00	0.00	0.00	0.00	0.16	0.00	0.00	0.00	0.00	0.16	0.00	0.00	0.00	0.00
Air	Sabatier	Water Outlet NC Solenoid	0.47	0.00	0.00	0.00	0.00	0.47	0.00	0.00	0.00	0.00	0.48	0.00	0.00	0.00	0.00	0.48	0.00	0.00	0.00	0.00	0.48	0.00	0.00	0.00	0.00
Air	Sabatier	Rotary Water Separator Assembly	4.04	0.00	22.66	22.66	0.00	4.04	0.00	22.66	22.66	0.00	4.10	0.00	22.93	22.93	0.00	4.08	0.00	22.84	22.84	0.00	4.07	0.00	22.80	22.80	0.00
Air	Sabatier	Sabatier Reactor Assembly	2.52	0.00	3.24	3.24	0.00	2.52	0.00	3.24	3.24	0.00	2.56	0.00	3.28	3.28	0.00	2.55	0.00	3.26	3.26	0.00	2.54	0.00	3.26	3.26	0.00
Air	Sabatier	Structure (A/R)	9.15	0.00	0.00	0.00	0.00	9.15	0.00	0.00	0.00	0.00	9.30	0.00	0.00	0.00	0.00	9.25	0.00	0.00	0.00	0.00	9.23	0.00	0.00	0.00	0.00
Air	Sabatier	Miscellaneous Hardware (clamps, bolts, etc.) (A/R)	1.77	0.00	0.00	0.00	0.00	1.77	0.00	0.00	0.00	0.00	1.77	0.00	0.00	0.00	0.00	1.77	0.00	0.00	0.00	0.00	1.77	0.00	0.00	0.00	0.00
Air	Sabatier	Air Cooling NC Solenoid	0.63	0.00	0.00	0.00	0.00	0.63	0.00	0.00	0.00	0.00	0.64	0.00	0.00	0.00	0.00	0.64	0.00	0.00	0.00	0.00	0.63	0.00	0.00	0.00	0.00
Air	Sabatier	Air Inlet Filter	0.11	0.00	0.00	0.00	0.00	0.11	0.00	0.00	0.00	0.00	0.11	0.00	0.00	0.00	0.00	0.11	0.00	0.00	0.00	0.00	0.11	0.00	0.00	0.00	0.00
Air	Sabatier	Air Sabatier Orifice	0.05	0.00	0.00	0.00	0.00	0.05	0.00	0.00	0.00	0.00	0.05	0.00	0.00	0.00	0.00	0.05	0.00	0.00	0.00	0.00	0.05	0.00	0.00	0.00	0.00
Air	Sabatier	Heat Exchanger Inlet Duct	0.10	0.00	0.00	0.00	0.00	0.10	0.00	0.00	0.00	0.00	0.11	0.00	0.00	0.00	0.00	0.11	0.00	0.00	0.00	0.00	0.11	0.00	0.00	0.00	0.00
Air	Sabatier	Heat Exchanger Outlet Duct	0.10	0.00	0.00	0.00	0.00	0.10	0.00	0.00	0.00	0.00	0.11	0.00	0.00	0.00	0.00	0.11	0.00	0.00	0.00	0.00	0.11	0.00	0.00	0.00	0.00

Supplementary Table 3. ESM parameters for inventory items broken down by system. Parameters are mass M [kg], volume V [m^3], power P [kW], cooling C [kW], crew-time CT [h][11,13]. ESM values correspond to a single sortie $S_{num} = 1$ for the scenarios described in Supplementary Table 2. (ORU = Orbital Replacement Unit)

			A					B					C					D					E				
**System**	**Subsystem**	**Item**	**M**	**V**	**P**	**C**	**CT**	**M**	**V**	**P**	**C**	**CT**	**M**	**V**	**P**	**C**	**CT**	**M**	**V**	**P**	**C**	**CT**	**M**	**V**	**P**	**C**	**CT**
Air	Sabatier	Reactor Inlet Duct	0.21	0.00	0.00	0.00	0.00	0.21	0.00	0.00	0.00	0.00	0.21	0.00	0.00	0.00	0.00	0.21	0.00	0.00	0.00	0.00	0.21	0.00	0.00	0.00	0.00
Air	Sabatier	Reactor Outlet Duct	0.10	0.00	0.00	0.00	0.00	0.10	0.00	0.00	0.00	0.00	0.11	0.00	0.00	0.00	0.00	0.11	0.00	0.00	0.00	0.00	0.11	0.00	0.00	0.00	0.00
Air	Sabatier	Tubing (A/R)	0.68	0.00	0.00	0.00	0.00	0.68	0.00	0.00	0.00	0.00	0.69	0.00	0.00	0.00	0.00	0.69	0.00	0.00	0.00	0.00	0.69	0.00	0.00	0.00	0.00
Air	Sabatier	Harnesses	11.45	0.00	0.00	0.00	0.00	11.45	0.00	0.00	0.00	0.00	11.45	0.00	0.00	0.00	0.00	11.45	0.00	0.00	0.00	0.00	11.45	0.00	0.00	0.00	0.00
Air	Sabatier	Valves + Sensors' total power	0.00	0.00	7.37	7.37	0.00	0.00	0.00	7.37	7.37	0.00	0.00	0.00	7.76	7.76	0.00	0.00	0.00	7.63	7.63	0.00	0.00	0.00	7.57	7.57	0.00
Air	Sabatier	Mechanical Compressor ORU	18.88	0.00	45.32	45.32	0.00	18.88	0.00	45.32	45.32	0.00	19.18	0.00	45.86	45.86	0.00	19.09	0.00	45.69	45.69	0.00	19.04	0.00	45.60	45.60	0.00
Air	Sabatier	Compressor Manifold Assembly	4.62	0.00	0.00	0.00	0.00	4.62	0.00	0.00	0.00	0.00	4.69	0.00	0.00	0.00	0.00	4.67	0.00	0.00	0.00	0.00	4.65	0.00	0.00	0.00	0.00
Air	Sabatier	Controller Assembly	28.59	0.00	55.00	55.00	0.00	28.59	0.00	55.00	55.00	0.00	28.59	0.00	55.00	55.00	0.00	28.59	0.00	55.00	55.00	0.00	28.59	0.00	55.00	55.00	0.00
Air	Sabatier	CO_2 Accumulator	10.03	0.01	0.00	0.00	0.00	10.03	0.01	0.00	0.00	0.00	10.19	0.01	0.00	0.00	0.00	10.14	0.01	0.00	0.00	0.00	10.11	0.01	0.00	0.00	0.00
Air	O_2-gen	Deionizing Bed ORU (Inlet)	7.79	0.01	0.00	0.00	0.00	7.79	0.01	0.00	0.00	0.00	8.19	0.01	0.00	0.00	0.00	8.07	0.01	0.00	0.00	0.00	8.00	0.01	0.00	0.00	0.00
Air	O_2-gen	Deionizing Bed ORU (Recirculating)	7.79	0.01	0.00	0.00	0.00	7.79	0.01	0.00	0.00	0.00	8.19	0.01	0.00	0.00	0.00	8.07	0.01	0.00	0.00	0.00	8.00	0.01	0.00	0.00	0.00
Air	O_2-gen	Oxygen/Water ORU	36.40	0.02	0.00	0.00	0.00	36.40	0.02	0.00	0.00	0.00	37.01	0.03	0.00	0.00	0.00	36.82	0.03	0.00	0.00	0.00	36.72	0.02	0.00	0.00	0.00
Air	O_2-gen	Pump ORU	6.46	0.01	23.33	23.33	0.00	6.46	0.01	23.33	23.33	0.00	6.56	0.01	24.53	24.53	0.00	6.53	0.01	24.15	24.15	0.00	6.51	0.01	23.96	23.96	0.00
Air	O_2-gen	Oxygen Phase Separator ORU	21.83	0.01	0.00	0.00	0.00	21.83	0.01	0.00	0.00	0.00	22.20	0.01	0.00	0.00	0.00	22.08	0.01	0.00	0.00	0.00	22.02	0.01	0.00	0.00	0.00
Air	O_2-gen	Hydrogen ORU	95.46	0.05	30.78	30.78	0.00	95.46	0.05	30.78	30.78	0.00	97.07	0.05	32.37	32.37	0.00	96.56	0.05	31.87	31.87	0.00	96.30	0.05	31.61	31.61	0.00
Air	O_2-gen	Hydrogen Sensor ORU	4.59	0.00	0.00	0.00	0.00	4.59	0.00	0.00	0.00	0.00	4.59	0.00	0.00	0.00	0.00	4.59	0.00	0.00	0.00	0.00	4.59	0.00	0.00	0.00	0.00
Air	O_2-gen	Process Controller	40.09	0.14	148.00	148.00	0.00	40.09	0.14	148.00	148.00	0.00	40.09	0.14	148.00	148.00	0.00	40.09	0.14	148.00	148.00	0.00	40.09	0.14	148.00	148.00	0.00
Air	O_2-gen	Power Supply Module (PSM)	13.45	0.02	1069.25	0.00	0.00	13.45	0.02	1069.25	0.00	0.00	14.14	0.02	1124.36	562.18	0.00	13.92	0.02	1106.94	553.47	0.00	13.81	0.02	1098.05	549.02	0.00
Air	Fire-det-sup	Fire Detection Assembly	1.50	0.00	1.48	1.48	0.03	1.50	0.00	1.48	1.48	0.03	1.50	0.00	1.48	1.48	0.03	1.50	0.00	1.48	1.48	0.08	1.50	0.00	1.48	1.48	0.89
Air	Fire-det-sup	Portable Fire Extinguisher	6.80	0.04	0.00	0.00	0.00	6.80	0.04	0.00	0.00	0.00	6.80	0.04	0.00	0.00	0.00	6.80	0.04	0.00	0.00	0.00	6.80	0.04	0.00	0.00	0.00
Air	ACO_2R	Regenerator 1	45.30	0.17	397.00	397.00	0.00	45.30	0.17	397.00	397.00	0.00	45.30	0.17	397.00	397.00	0.00	45.30	0.17	397.00	397.00	0.00	45.30	0.17	397.00	397.00	0.00
Air	ACO_2R	Metox Canisters	136.00	0.06	0.00	0.00	0.00	136.00	0.06	0.00	0.00	0.00	136.00	0.06	0.00	0.00	0.00	136.00	0.06	0.00	0.00	0.00	136.00	0.06	0.00	0.00	0.00
Air	TCCS-ISS	Activated Charcoal Bed	4.63	0.01	0.00	0.00	0.00	4.63	0.01	0.00	0.00	0.00	4.63	0.01	0.00	0.00	0.00	12.87	0.03	0.00	0.00	0.00	139.02	0.29	0.00	0.00	0.00
Air	TCCS-ISS	Blower Assembly	2.49	0.00	30.39	30.39	0.00	2.49	0.00	30.39	30.39	0.00	2.49	0.00	30.39	30.39	0.00	2.49	0.00	30.39	30.39	0.00	2.49	0.00	30.39	30.39	0.00
Air	TCCS-ISS	Flow Meter Assembly	1.10	0.00	11.50	11.50	0.00	1.10	0.00	11.50	11.50	0.00	1.10	0.00	11.50	11.50	0.00	1.10	0.00	11.50	11.50	0.00	1.10	0.00	11.50	11.50	0.00
Air	TCCS-ISS	Catalytic Oxidizer Assembly	8.46	0.02	92.19	92.19	0.00	8.46	0.02	92.19	92.19	0.00	8.46	0.02	92.19	92.19	0.00	8.46	0.02	92.19	92.19	0.00	8.46	0.02	92.19	92.19	0.00
Air	TCCS-ISS	LiOH Sorbent Bed Assembly	0.31	0.00	0.00	0.00	0.00	0.31	0.00	0.00	0.00	0.00	0.31	0.00	0.00	0.00	0.00	0.87	0.00	0.00	0.00	0.00	9.43	0.02	0.00	0.00	0.00
Air	TCCS-ISS	Electrical Interface Assembly	4.50	0.00	7.60	7.60	0.00	4.50	0.00	7.60	7.60	0.00	4.50	0.00	7.60	7.60	0.00	4.50	0.00	7.60	7.60	0.00	4.50	0.00	7.60	7.60	0.00
Waste	PMWC	Aluminum Compaction Cylinder	9.40	0.02	0.00	0.00	180.00	9.40	0.02	0.00	0.00	180.00	9.41	0.02	0.00	0.00	180.00	9.41	0.02	0.00	0.00	500.00	9.41	0.02	0.00	0.00	5400.00
Waste	PMWC	Band-type Heating Unit	0.00	0.00	136.34	0.00	0.00	0.00	0.00	136.34	0.00	0.00	0.00	0.00	162.58	0.00	0.00	0.00	0.00	162.39	0.00	0.00	0.00	0.00	162.29	0.00	0.00
Waste	PMWC	Lightweight, Oil-Less, Compressor/Vacuum Pump	0.00	0.00	2.71	0.00	0.00	0.00	0.00	2.71	0.00	0.00	0.00	0.00	3.23	0.00	0.00	0.00	0.00	3.22	0.00	0.00	0.00	0.00	3.22	0.00	0.00
Waste	PMWC	Temperature Sensor	0.22	0.00	0.00	0.00	0.00	0.22	0.00	0.00	0.00	0.00	0.22	0.00	0.00	0.00	0.00	0.22	0.00	0.00	0.00	0.00	0.22	0.00	0.00	0.00	0.00
Waste	PMWC	Pressure Sensor	0.30	0.00	0.33	0.00	0.00	0.30	0.00	0.33	0.00	0.00	0.30	0.00	0.33	0.00	0.00	0.30	0.00	0.33	0.00	0.00	0.30	0.00	0.33	0.00	0.00
Waste	PMWC	Housing + Mounting Equipment	23.86	0.48	0.00	0.00	0.00	23.86	0.48	0.00	0.00	0.00	23.89	0.48	0.00	0.00	0.00	23.89	0.48	0.00	0.00	0.00	23.89	0.48	0.00	0.00	0.00
Waste	PMWC	Condensing Heat Exchanger	1.91	0.00	0.00	0.00	0.00	1.91	0.00	0.00	0.00	0.00	2.86	0.01	0.00	0.00	0.00	2.86	0.01	0.00	0.00	0.00	2.85	0.01	0.00	0.00	0.00
Waste	PMWC	Cooling system	0.00	0.00	0.00	0.00	0.00	0.00	0.00	0.00	0.00	0.00	0.00	0.00	145.14	145.14	0.00	0.00	0.00	144.97	144.97	0.00	0.00	0.00	144.89	144.89	0.00
Waste	Waste-storage	Low Density PolyEthylene Box	17.70	0.49	0.00	0.00	0.00	17.70	0.49	0.00	0.00	0.00	17.70	0.49	0.00	0.00	0.00	49.18	1.37	0.00	0.00	0.00	531.15	14.75	0.00	0.00	0.00

Supplementary Table 3. ESM parameters for inventory items broken down by system. Parameters are mass M [kg], volume V [m^3], power P [kW], cooling C [kW], crew-time CT [h][11,13]. ESM values correspond to a single sortie $S_{num} = 1$ for the scenarios described in Supplementary Table 2. (ORU = Orbital Replacement Unit)

			A					B					C					D					E				
**System**	**Subsystem**	**Item**	**M**	**V**	**P**	**C**	**CT**	**M**	**V**	**P**	**C**	**CT**	**M**	**V**	**P**	**C**	**CT**	**M**	**V**	**P**	**C**	**CT**	**M**	**V**	**P**	**C**	**CT**
Waste	Waste-col	Commode/Urinal	58.40	0.30	0.00	0.00	29.59	58.40	0.30	0.00	0.00	29.59	58.40	0.30	0.00	0.00	29.59	58.40	0.30	0.00	0.00	82.19	58.40	0.30	0.00	0.00	887.67
Waste	Waste-col	Fan	0.00	0.00	102.00	102.00	0.00	0.00	0.00	102.00	102.00	0.00	0.00	0.00	102.00	102.00	0.00	0.00	0.00	102.00	102.00	0.00	0.00	0.00	102.00	102.00	0.00
Waste	Waste-col	Urine Separator	0.00	0.00	125.00	125.00	0.00	0.00	0.00	125.00	125.00	0.00	0.00	0.00	125.00	125.00	0.00	0.00	0.00	125.00	125.00	0.00	0.00	0.00	125.00	125.00	0.00
Waste	Waste-col	Urine Vent Heater	0.00	0.00	14.00	14.00	0.00	0.00	0.00	14.00	14.00	0.00	0.00	0.00	14.00	14.00	0.00	0.00	0.00	14.00	14.00	0.00	0.00	0.00	14.00	14.00	0.00
Waste	Waste-col	Fecal Bags	19.64	0.22	0.00	0.00	0.00	19.64	0.22	0.00	0.00	0.00	19.64	0.22	0.00	0.00	0.00	54.55	0.61	0.00	0.00	0.00	589.09	6.58	0.00	0.00	0.00
Waste	Waste-col	Wipes, Dry	6.55	0.09	0.00	0.00	0.00	6.55	0.09	0.00	0.00	0.00	6.55	0.09	0.00	0.00	0.00	18.18	0.25	0.00	0.00	0.00	196.36	2.65	0.00	0.00	0.00
Waste	Waste-col	Wipes, Wet	10.23	0.05	0.00	0.00	0.00	10.23	0.05	0.00	0.00	0.00	10.23	0.05	0.00	0.00	0.00	28.41	0.15	0.00	0.00	0.00	306.82	1.59	0.00	0.00	0.00
Waste	Waste-col	Wipes, Toilet Tissue	3.53	0.04	0.00	0.00	0.00	3.53	0.04	0.00	0.00	0.00	3.53	0.04	0.00	0.00	0.00	9.82	0.12	0.00	0.00	0.00	106.04	1.29	0.00	0.00	0.00
Waste	Waste-col	Gloves	5.60	0.03	0.00	0.00	0.00	5.60	0.03	0.00	0.00	0.00	5.60	0.03	0.00	0.00	0.00	15.55	0.07	0.00	0.00	0.00	167.89	0.76	0.00	0.00	0.00
Waste	Waste-col	Fecal Bags Odor Lids	29.45	0.28	0.00	0.00	0.00	29.45	0.28	0.00	0.00	0.00	29.45	0.28	0.00	0.00	0.00	81.82	0.77	0.00	0.00	0.00	883.64	8.26	0.00	0.00	0.00
Waste	Waste-col	Fecal Collection Canisters	35.06	0.46	0.00	0.00	0.00	35.06	0.46	0.00	0.00	0.00	35.06	0.46	0.00	0.00	0.00	97.40	1.28	0.00	0.00	0.00	1051.95	13.77	0.00	0.00	0.00
Waste	Waste-col	Fecal collection Canisters lids	17.53	0.07	0.00	0.00	0.00	17.53	0.07	0.00	0.00	0.00	17.53	0.07	0.00	0.00	0.00	48.70	0.20	0.00	0.00	0.00	525.97	2.15	0.00	0.00	0.00
Waste	Waste-col	Urine Prefilters	30.68	0.17	0.00	0.00	0.00	30.68	0.17	0.00	0.00	0.00	30.68	0.17	0.00	0.00	0.00	85.23	0.46	0.00	0.00	0.00	920.45	5.01	0.00	0.00	0.00
Waste	Waste-col	Urine Filters	4.38	0.04	0.00	0.00	0.00	4.38	0.04	0.00	0.00	0.00	4.38	0.04	0.00	0.00	0.00	12.18	0.10	0.00	0.00	0.00	131.49	1.06	0.00	0.00	0.00
Waste	Waste-col	Urine Funnels	0.00	0.00	0.00	0.00	0.00	0.00	0.00	0.00	0.00	0.00	1.16	0.01	0.00	0.00	0.00	1.16	0.01	0.00	0.00	0.00	1.16	0.01	0.00	0.00	0.00
Waste	Waste-col	Flush Water Transfer Bags	5.61	0.08	0.00	0.00	0.00	5.61	0.08	0.00	0.00	0.00	5.61	0.08	0.00	0.00	0.00	15.58	0.22	0.00	0.00	0.00	168.31	2.35	0.00	0.00	0.00
Waste	TCCS-ISS-x3	Activated charcoal bed	9.27	0.02	0.00	0.00	0.00	9.27	0.02	0.00	0.00	0.00	9.27	0.02	0.00	0.00	0.00	25.74	0.05	0.00	0.00	0.00	278.04	0.57	0.00	0.00	0.00
Waste	TCCS-ISS-x4	Blower Assembly	4.97	0.01	60.79	60.79	0.00	4.97	0.01	60.79	60.79	0.00	4.97	0.01	60.79	60.79	0.00	4.97	0.01	60.79	60.79	0.00	4.97	0.01	60.79	60.79	0.00
Waste	TCCS-ISS-x5	Flow Meter Assembly	2.20	0.00	23.00	23.00	0.00	2.20	0.00	23.00	23.00	0.00	2.20	0.00	23.00	23.00	0.00	2.20	0.00	23.00	23.00	0.00	2.20	0.00	23.00	23.00	0.00
Waste	TCCS-ISS-x6	Catalytic Oxidizer Assembly	16.91	0.04	184.38	184.38	0.00	16.91	0.04	184.38	184.38	0.00	16.91	0.04	184.38	184.38	0.00	16.91	0.04	184.38	184.38	0.00	16.91	0.04	184.38	184.38	0.00
Waste	TCCS-ISS-x7	LiOH Sorbent Bed Assembly	0.63	0.00	0.00	0.00	0.00	0.63	0.00	0.00	0.00	0.00	0.63	0.00	0.00	0.00	0.00	1.75	0.00	0.00	0.00	0.00	18.86	0.04	0.00	0.00	0.00
Waste	TCCS-ISS-x8	Electrical Interface Assembly	9.00	0.01	15.20	15.20	0.00	9.00	0.01	15.20	15.20	0.00	9.00	0.01	15.20	15.20	0.00	9.00	0.01	15.20	15.20	0.00	9.00	0.01	15.20	15.20	0.00
Water	H_2O-rec	MLS Filter ORU	3.32	0.00	0.00	0.00	0.00	3.32	0.00	0.00	0.00	0.00	3.96	0.01	0.00	0.00	0.00	10.97	0.02	0.00	0.00	0.00	118.21	0.17	0.00	0.00	0.00
Water	H_2O-rec	Particulate Filter ORU	17.22	0.04	0.00	0.00	0.00	17.22	0.04	0.00	0.00	0.00	20.54	0.05	0.00	0.00	0.00	56.83	0.14	0.00	0.00	0.00	612.48	1.56	0.00	0.00	0.00
Water	H_2O-rec	Multifiltration Bed #1 + #2 ORUs	0.00	0.00	0.00	0.00	0.00	0.00	0.00	0.00	0.00	0.00	167.74	0.22	0.00	0.00	0.00	464.08	0.60	0.00	0.00	0.00	5001.86	6.51	0.00	0.00	0.00
Water	H_2O-rec	Sensor ORU	3.64	0.01	2.13	2.13	0.00	3.64	0.01	2.13	2.13	0.00	3.64	0.01	2.13	2.13	0.00	3.64	0.01	2.13	2.13	0.00	3.64	0.01	2.13	2.13	0.00
Water	H_2O-rec	Piping	5.37	0.01	0.00	0.00	0.00	5.37	0.01	0.00	0.00	0.00	0.74	0.00	0.00	0.00	0.00	0.74	0.00	0.00	0.00	0.00	0.74	0.00	0.00	0.00	0.00
Water	H_2O-rec	Pump/MLS ORU	30.28	0.09	51.17	51.17	0.00	30.28	0.09	51.17	51.17	0.00	18.42	0.06	19.62	19.62	0.00	18.40	0.06	19.54	19.54	0.00	18.38	0.06	19.50	19.50	0.00
Water	H_2O-rec	Catalytic Reactor + Preheater ORU	0.00	0.00	0.00	0.00	0.00	0.00	0.00	0.00	0.00	0.00	0.00	0.00	107.90	107.90	0.00	0.00	0.00	107.47	107.47	0.00	0.00	0.00	107.25	107.25	0.00
Water	H_2O-rec	Oxygen Filter	0.00	0.00	0.00	0.00	0.00	0.00	0.00	0.00	0.00	0.00	0.00	0.00	0.00	0.00	0.00	0.00	0.00	0.00	0.00	0.00	0.00	0.00	0.00	0.00	0.00
Water	H_2O-rec	Microbial Check Valve	0.00	0.00	0.00	0.00	0.00	0.00	0.00	0.00	0.00	0.00	5.16	0.01	0.00	0.00	0.00	5.13	0.01	0.00	0.00	0.00	5.12	0.01	0.00	0.00	0.00
Water	H_2O-rec	Gas Separator ORU	43.11	0.09	132.33	132.33	0.00	43.11	0.09	132.33	132.33	0.00	26.24	0.06	50.73	50.73	0.00	26.20	0.06	50.53	50.53	0.00	26.17	0.06	50.43	50.43	0.00
Water	H_2O-rec	Hygiene H_2O Tank	95.90	0.18	7.05	7.05	0.00	95.90	0.18	7.05	7.05	0.00	58.36	0.11	4.72	4.72	0.00	58.27	0.11	4.71	4.71	0.00	58.22	0.11	4.71	4.71	0.00
Water	H_2O-rec	Product H_2O Tank	53.97	0.19	7.85	7.85	0.00	53.97	0.19	7.85	7.85	0.00	32.84	0.12	5.25	5.25	0.00	32.79	0.12	5.24	5.24	0.00	32.76	0.12	5.24	5.24	0.00
Water	H_2O-rec	Process Controller	36.91	0.08	156.18	156.18	0.00	36.91	0.08	156.18	156.18	0.00	36.91	0.08	156.18	156.18	0.00	36.91	0.08	156.18	156.18	0.00	36.91	0.08	156.18	156.18	0.00
Water	H_2O-rec	Reactor Health Sensor	8.64	0.04	4.72	4.72	0.00	8.64	0.04	4.72	4.72	0.00	8.64	0.04	4.72	4.72	0.00	8.64	0.04	4.72	4.72	0.00	8.64	0.04	4.72	4.72	0.00
Water	H_2O-rec	H_2O Delivery System	42.97	0.11	2.88	2.88	0.00	42.97	0.11	2.88	2.88	0.00	26.15	0.06	1.93	1.93	0.00	26.11	0.06	1.93	1.93	0.00	26.09	0.06	1.92	1.92	0.00

Supplementary Table 3. ESM parameters for inventory items broken down by system. Parameters are mass M [kg], volume V [m^3], power P [kW], cooling C [kW], crew-time CT [h][11,13]. ESM values correspond to a single sortie $S_{num} = 1$ for the scenarios described in Supplementary Table 2. (ORU = Orbital Replacement Unit)

			A					B					C					D					E				
**System**	**Subsystem**	**Item**	**M**	**V**	**P**	**C**	**CT**	**M**	**V**	**P**	**C**	**CT**	**M**	**V**	**P**	**C**	**CT**	**M**	**V**	**P**	**C**	**CT**	**M**	**V**	**P**	**C**	**CT**
Water	WRS	Ion Exchange Bed	2.78	0.00	0.00	0.00	0.00	2.78	0.00	0.00	0.00	0.00	2.78	0.00	0.00	0.00	0.00	7.70	0.01	0.00	0.00	0.00	83.02	0.11	0.00	0.00	0.00
Water	Urine-proc	Pressure Control + Pump (PCPA)	27.14	0.04	5.73	5.73	0.00	27.14	0.04	5.73	5.73	0.00	33.73	0.05	9.83	9.83	0.00	33.64	0.05	9.77	9.77	0.00	33.60	0.05	9.75	9.75	0.00
Water	Urine-proc	Fluid Control + Pump (FCPA)	27.63	0.04	8.30	8.30	0.00	27.63	0.04	8.30	8.30	0.00	34.35	0.05	14.26	14.26	0.00	34.26	0.05	14.18	14.18	0.00	34.21	0.05	14.14	14.14	0.00
Water	Urine-proc	Recycle Filter Tank (RFTA)	11.55	0.06	0.00	0.00	0.00	11.55	0.06	0.00	0.00	0.00	14.35	0.08	0.00	0.00	0.00	14.32	0.08	0.00	0.00	0.00	14.30	0.08	0.00	0.00	0.00
Water	Urine-proc	Wastewater Storage Tank Assembly (WSTA)	28.95	0.02	0.08	0.08	0.00	28.95	0.02	0.08	0.08	0.00	35.98	0.03	0.14	0.14	0.00	35.89	0.03	0.14	0.14	0.00	35.84	0.03	0.14	0.14	0.00
Water	Urine-proc	Distillation Assembly (DA)	45.81	0.09	79.35	79.35	0.00	45.81	0.09	79.35	79.35	0.00	56.94	0.11	136.22	136.22	0.00	56.79	0.11	135.46	135.46	0.00	56.72	0.11	135.08	135.08	0.00
Water	Urine-proc	Separator Plumbing Assembly (SPA)	9.87	0.02	0.00	0.00	0.00	9.87	0.02	0.00	0.00	0.00	12.27	0.02	0.00	0.00	0.00	12.24	0.02	0.00	0.00	0.00	12.22	0.02	0.00	0.00	0.00
Water	Urine-proc	Power Module (Included in FCA)	0.00	0.00	0.00	0.00	0.00	0.00	0.00	0.00	0.00	0.00	0.00	0.00	0.00	0.00	0.00	0.00	0.00	0.00	0.00	0.00	0.00	0.00	0.00	0.00	0.00
Water	Urine-proc	Firmware Controller Assembly (Data Module, Power Module)	24.09	0.03	150.09	150.09	0.00	24.09	0.03	150.09	150.09	0.00	24.09	0.03	150.09	150.09	0.00	24.09	0.03	150.09	150.09	0.00	24.09	0.03	150.09	150.09	0.00
Water	Urine-proc	Piping	7.55	0.01	0.00	0.00	0.00	7.55	0.01	0.00	0.00	0.00	9.38	0.02	0.00	0.00	0.00	9.36	0.02	0.00	0.00	0.00	9.34	0.02	0.00	0.00	0.00
Water	Volatile-rem	Catalytic Reactor + Preheater ORU	0.00	0.00	90.48	90.48	0.00	0.00	0.00	90.48	90.48	0.00	0.00	0.00	90.48	90.48	0.00	0.00	0.00	90.48	90.48	0.00	0.00	0.00	90.48	90.48	0.00
Water	Volatile-rem	Gas Separator ORU	24.54	0.05	42.54	42.54	0.00	24.54	0.05	42.54	42.54	0.00	24.54	0.05	42.54	42.54	0.00	24.54	0.05	42.54	42.54	0.00	24.54	0.05	42.54	42.54	0.00
Water	Volatile-rem	Oxygen Filter	0.00	0.00	0.00	0.00	0.00	0.00	0.00	0.00	0.00	0.00	0.00	0.00	0.00	0.00	0.00	0.00	0.00	0.00	0.00	0.00	0.00	0.00	0.00	0.00	0.00
Water	Volatile-rem	Piping	1.26	0.00	0.00	0.00	0.00	1.26	0.00	0.00	0.00	0.00	1.26	0.00	0.00	0.00	0.00	1.26	0.00	0.00	0.00	0.00	1.26	0.00	0.00	0.00	0.00
Water	Tank	Product H_2O Tank	101.12	0.36	13.65	13.65	0.00	101.12	0.36	13.65	13.65	0.00	84.82	0.30	11.64	11.64	0.00	137.09	0.49	18.07	18.07	0.00	937.43	3.33	116.48	116.48	0.00
Water	Tank	H_2O Stored	256.99	0.00	0.00	0.00	0.00	256.99	0.00	0.00	0.00	0.00	205.56	0.00	0.00	0.00	0.00	370.55	0.00	0.00	0.00	0.00	2897.03	0.00	0.00	0.00	0.00
Food	Food-storage	Packaging	0.00	0.00	0.00	0.00	0.00	0.00	0.00	0.00	0.00	0.00	990.00	0.00	0.00	0.00	0.00	2707.39	0.00	0.00	0.00	0.00	29004.85	0.00	0.00	0.00	0.00
Food	Food-storage	Lockers/Storage	218.86	3.05	0.00	0.00	0.00	218.86	3.05	0.00	0.00	0.00	242.76	3.39	0.00	0.00	0.00	663.88	9.26	0.00	0.00	0.00	7112.33	99.24	0.00	0.00	0.00
Food	Food-processing	Rehydration Unit and Conduction Oven	36.30	0.09	960.00	960.00	0.00	36.30	0.09	960.00	960.00	0.00	36.30	0.09	10.00	10.00	0.00	36.30	0.09	10.00	10.00	0.00	36.30	0.09	10.00	10.00	0.00
Thermal	CCAA	Inlet ORU	0.00	0.00	312.64	312.64	0.00	0.00	0.00	312.64	312.64	0.00	0.00	0.00	299.23	299.23	0.00	0.00	0.00	299.08	299.08	0.00	0.00	0.00	299.00	299.00	0.00
Thermal	CCAA	Condensing Heat Exchanger	37.69	0.07	0.00	0.00	0.00	37.69	0.07	0.00	0.00	0.00	34.91	0.07	0.00	0.00	0.00	34.90	0.07	0.00	0.00	0.00	34.89	0.07	0.00	0.00	0.00
Thermal	CCAA	Water Separator	6.41	0.04	10.19	10.19	0.00	6.41	0.04	10.19	10.19	0.00	5.31	0.04	3.38	3.38	0.00	5.30	0.04	3.35	3.35	0.00	5.30	0.04	3.34	3.34	0.00
Thermal	CCAA	Temp. Control + Check Valve	4.09	0.03	0.06	0.06	0.00	4.09	0.03	0.06	0.06	0.00	3.89	0.03	0.06	0.06	0.00	3.89	0.03	0.06	0.06	0.00	3.89	0.03	0.06	0.06	0.00
Thermal	CCAA	Electrical Interface Box (EIB)	4.10	0.01	8.00	8.00	0.00	4.10	0.01	8.00	8.00	0.00	4.10	0.01	8.00	8.00	0.00	4.10	0.01	8.00	8.00	0.00	4.10	0.01	8.00	8.00	0.00
Thermal	CCAA	Temp. Sensor	0.24	0.00	0.01	0.01	0.00	0.24	0.00	0.01	0.01	0.00	0.24	0.00	0.01	0.01	0.00	0.24	0.00	0.01	0.01	0.00	0.24	0.00	0.01	0.01	0.00
Thermal	CCAA	Liquid Sensor	0.47	0.00	0.01	0.01	0.00	0.47	0.00	0.01	0.01	0.00	0.47	0.00	0.01	0.01	0.00	0.47	0.00	0.01	0.01	0.00	0.47	0.00	0.01	0.01	0.00
Thermal	CCAA	Fan Delta P Sensor	0.40	0.00	0.20	0.20	0.00	0.40	0.00	0.20	0.20	0.00	0.40	0.00	0.20	0.20	0.00	0.40	0.00	0.20	0.20	0.00	0.40	0.00	0.20	0.20	0.00
Thermal	CCAA	Pressure Sensor	0.30	0.00	0.20	0.20	0.00	0.30	0.00	0.20	0.20	0.00	0.30	0.00	0.20	0.20	0.00	0.30	0.00	0.20	0.20	0.00	0.30	0.00	0.20	0.20	0.00
Thermal	Atmos-cont	HEPA Filter Element	49.22	0.19	0.00	0.00	0.99	49.22	0.19	0.00	0.00	0.99	49.22	0.19	0.00	0.00	0.99	49.22	0.19	0.00	0.00	2.74	49.22	0.19	0.00	0.00	29.59
Thermal	Atmos-cont	Catalytic Filter Element	54.00	0.08	0.00	0.00	0.00	54.00	0.08	0.00	0.00	0.00	54.00	0.08	0.00	0.00	0.00	54.00	0.08	0.00	0.00	0.00	54.00	0.08	0.00	0.00	0.00
Thermal	Atmosphere-circ	IMV Fan	4.77	0.01	55.00	55.00	0.00	4.77	0.01	55.00	55.00	0.00	4.77	0.01	55.00	55.00	0.00	4.77	0.01	55.00	55.00	0.00	4.77	0.01	55.00	55.00	0.00
Thermal	Atmosphere-circ	IMV Valve	5.10	0.01	6.00	6.00	0.00	5.10	0.01	6.00	6.00	0.00	5.10	0.01	6.00	6.00	0.00	5.10	0.01	6.00	6.00	0.00	5.10	0.01	6.00	6.00	0.00
Thermal	AAA	Avionics Air Assembly	12.40	0.03	175.00	175.00	0.00	12.40	0.03	175.00	175.00	0.00	12.40	0.03	175.00	175.00	0.00	12.40	0.03	175.00	175.00	0.00	12.40	0.03	175.00	175.00	0.00
Thermal	ITCS	ITCS	211.83	0.36	2585.10	2585.10	0.00	211.83	0.36	2585.10	2585.10	0.00	211.83	0.36	2585.10	2585.10	0.00	212.49	0.36	2595.44	2595.44	0.00	217.23	0.36	2669.94	2669.94	0.00

Supplementary Table 4. Estimation of inventory items into exemplar classes broken down by system for each scenario described in Supplementary Table 2. Classes include: Structural Metal, Plastic, Electronics, Fabric, Glass, Rubber, Ceramics, Gas, Biomass, Water, Other. (ORU = Orbital Replacement Unit)

System	Subsystem	Item	Structural Metal	Plastic	Electronics	Fabric	Glass	Rubber	Ceramics	Gas	Biomass	Water	Other
Air	APC	Vent/Relief Valve	1	0	0	0	0	0	0	0	0	0	0
Air	APC	Pressure Control Panel	0.2	0.1	0.7	0	0	0	0	0	0	0	0
Air	APC	Manual Pressure Equalization Valve	1	0	0	0	0	0	0	0	0	0	0
Air	APC	Positive Pressure Relief Valve	1	0	0	0	0	0	0	0	0	0	0
Air	APC	Negative Pressure Relief Valve	1	0	0	0	0	0	0	0	0	0	0
Air	APC	Nitrogen Interface Assembly	1	0	0	0	0	0	0	0	0	0	0
Air	APC	Vacuum Access Jumper 5-ft	0	0	1	0	0	0	0	0	0	0	0
Air	APC	Vacuum Access Jumper 35-ft	0	0	1	0	0	0	0	0	0	0	0
Air	ACMA	Verification Gas Assembly	0.5	0	0	0	0	0	0	0.5	0	0	0
Air	ACMA	Mass Spectrometer	0.5	0	0.5	0	0	0	0	0	0	0	0
Air	ACMA	Sample Pump	1	0	0	0	0	0	0	0	0	0	0
Air	ACMA	Sample Distributor	0.95	0	0	0.05	0	0	0	0	0	0	0
Air	ACMA	Data + Control	0.5	0	0.5	0	0	0	0	0	0	0	0
Air	ACMA	Low Volt. Power supply	0.7	0	0	0	0	0.1	0.1	0	0	0	0.1
Air	ACMA	Chassis	1	0	0	0	0	0	0	0	0	0	0
Air	ACMA	Inlet Valve Assembly	0.9	0	0	0.01	0	0	0	0	0	0.09	0
Air	ACMA	EMI Filter	0.98	0.02	0	0	0	0	0	0	0	0	0
Air	SDS	3-way Solenoid Valves	1	0	0	0	0	0	0	0	0	0	0
Air	SDS	Manual Valves	1	0	0	0	0	0	0	0	0	0	0
Air	SDS	Sample Probes	1	0	0	0	0	0	0	0	0	0	0
Air	CO_2-rem	Air Selector Valve	0.9	0	0	0	0	0	0	0.1	0	0	0
Air	CO_2-rem	Desiccant Bed	0.4	0	0	0	0	0	0.6	0	0	0	0
Air	CO_2-rem	Adsorbent Bed	0.5	0	0	0.2	0	0	0.3	0	0	0	0
Air	CO_2-rem	Air Check Valve	1	0	0	0	0	0	0	0	0	0	0
Air	CO_2-rem	Heater Controller	0.8	0	0.2	0	0	0	0	0	0	0	0
Air	CO_2-rem	Air Blower	1	0	0	0	0	0	0	0	0	0	0
Air	CO_2-rem	Pre-cooler	1	0	0	0	0	0	0	0	0	0	0
Air	CO_2-rem	Blower/Pre-cooler Motor Controller	0.6	0	0.4	0	0	0	0	0	0	0	0
Air	CO_2-rem	CO_2 Pump	0.99	0	0	0	0	0	0	0	0	0	0.01
Air	CO_2-rem	CO_2 Pump Motor Controller	0.3	0	0.7	0	0	0	0	0	0	0	0
Air	CO_2-rem	Temperature Sensor	0	0	0.6	0	0.4	0	0	0	0	0	0
Air	CO_2-rem	Differential Pressure Sensor	0.1	0.25	0.65	0	0	0	0	0	0	0	0
Air	CO_2-rem	Absolute Pressure Sensor	0.75	0	0	0	0	0	0.25	0	0	0	0
Air	CO_2-rem	Electrical Harness	0.9	0.05	0	0.05	0	0	0	0	0	0	0
Air	CO_2-rem	Plumbing	1	0	0	0	0	0	0	0	0	0	0
Air	CO_2-rem	Support Structure	1	0	0	0	0	0	0	0	0	0	0
Air	CO_2-rem	Fluid Disconnects	1	0	0	0	0	0	0	0	0	0	0
Air	CO_2-rem	Electronics Cold-Plate	1	0	0	0	0	0	0	0	0	0	0
Air	CO_2-rem	Electronics Interface Plate	1	0	0	0	0	0	0	0	0	0	0
Air	N_2	MD Shield Instl	1	0	0	0	0	0	0	0	0	0	0
Air	N_2	Multilayer Insulation Assembly-T #1	1	0	0	0	0	0	0	0	0	0	0
Air	N_2	Multilayer Insulation Assembly-T #2	1	0	0	0	0	0	0	0	0	0	0
Air	N_2	Primary Structure Assembly-HPG ORU	0.5	0	0	0	0	0	0	0.5	0	0	0
Air	N_2	Tank ORU Assembly	1	0	0	0	0	0	0	0	0	0	0
Air	N_2	Utilities Installation - O_2/N_2 Tank	0.5	0	0	0	0	0	0	0.5	0	0	0
Air	N_2	N_2 Bare Tank	0.9	0	0	0	0	0.05	0	0.05	0	0	0
Air	N_2	HPGA Fluid	0	0	0	0	0	0	0	0	0	1	0
Air	N_2	Handhold, top mounted	0	1	0	0	0	0	0	0	0	0	0
Air	N_2	Handrail 21.941 in custom	0	1	0	0	0	0	0	0	0	0	0
Air	N_2	Handrail, top mounted	0	1	0	0	0	0	0	0	0	0	0
Air	N_2	Grapple Fixture, flt releasable	0.5	0	0.5	0	0	0	0	0	0	0	0
Air	N_2	Accessories	0	0	0	0	0	0	0	0	0	0	0
Air	O_2	MD Shield Instl	1	0	0	0	0	0	0	0	0	0	0
Air	O_2	Multilayer Insulation Assembly-T #1	1	0	0	0	0	0	0	0	0	0	0
Air	O_2	Multilayer Insulation Assembly-T #2	1	0	0	0	0	0	0	0	0	0	0
Air	O_2	Primary Structure Assembly-HPG ORU	0.5	0	0	0	0	0	0	0.5	0	0	0
Air	O_2	Tank ORU Assembly	1	0	0	0	0	0	0	0	0	0	0
Air	O_2	Utilities Installation - O_2/N_2 Tank	0.5	0	0	0	0	0	0	0.5	0	0	0
Air	O_2	O_2 Bare Tank	0.95	0	0	0	0	0	0	0.05	0	0	0
Air	O_2	HPGA Fluid	0	0	0	0	0	0	0	0	0	1	0
Air	O_2	Handhold, top mounted	0	1	0	0	0	0	0	0	0	0	0
Air	O_2	Handrail 21.941 in custom	0	1	0	0	0	0	0	0	0	0	0
Air	O_2	Handrail, top mounted	0	1	0	0	0	0	0	0	0	0	0
Air	O_2	Grapple Fixture, flt releasable	0.5	0	0.5	0	0	0	0	0	0	0	0
Air	O_2	Accessories	0	0	0	0	0	0	0	0	0	0	0
Air	Sabatier	Condensing Heat Exchanger	0.9	0	0.1	0	0	0	0	0	0	0	0
Air	Sabatier	AAA Heat Exchanger	1	0	0	0	0	0	0	0	0	0	0
Air	Sabatier	ITCS Coolant Water Inlet QD	1	0	0	0	0	0	0	0	0	0	0
Air	Sabatier	ITCS Coolant Water Outlet QD	1	0	0	0	0	0	0	0	0	0	0
Air	Sabatier	Heat Exchanger Inlet Temp	1	0	0	0	0	0	0	0	0	0	0
Air	Sabatier	Heat Exchanger Outlet Temp	1	0	0	0	0	0	0	0	0	0	0
Air	Sabatier	Manifold, CO_2	0.95	0.05	0	0	0	0	0	0	0	0	0
Air	Sabatier	CO_2 Inlet Check Valve	0.9	0.1	0	0	0	0	0	0	0	0	0

Supplementary Table 4. Estimation of inventory items into exemplar classes broken down by system for each scenario described in Supplementary Table 2. Classes include: Structural Metal, Plastic, Electronics, Fabric, Glass, Rubber, Ceramics, Gas, Biomass, Water, Other. (ORU = Orbital Replacement Unit)

System	Subsystem	Item	Structural Metal	Plastic	Electronics	Fabric	Glass	Rubber	Ceramics	Gas	Biomass	Water	Other
Air	Sabatier	CO_2 Inlet Filter	1	0	0	0	0	0	0	0	0	0	0
Air	Sabatier	Pressure Sensor, CO_2 Inlet	0	0	0.6	0	0.4	0	0	0	0	0	0
Air	Sabatier	CO_2 Inlet QD	1	0	0	0	0	0	0	0	0	0	0
Air	Sabatier	CO_2 Inlet Regulator	0.9	0.05	0	0	0.05	0	0	0	0	0	0
Air	Sabatier	CO_2 Inlet NC Solenoid	1	0	0	0	0	0	0	0	0	0	0
Air	Sabatier	CO_2 Inlet Flow Control	0.85	0.05	0	0	0.1	0	0	0	0	0	0
Air	Sabatier	CO_2 Flow Control Orifice	1	0	0	0	0	0	0	0	0	0	0
Air	Sabatier	Delta P Sensor, Flow Sensor CO_2	0	0	0.6	0	0.4	0	0	0	0	0	0
Air	Sabatier	CO_2 Flow Meter Orifice	1	0	0	0	0	0	0	0	0	0	0
Air	Sabatier	Manifold, Hydrogen	0.95	0.05	0	0	0	0	0	0	0	0	0
Air	Sabatier	Water Outlet Quick Disconnect	1	0	0	0	0	0	0	0	0	0	0
Air	Sabatier	Hydrogen Inlet Check Valve	0.9	0.1	0	0	0	0	0	0	0	0	0
Air	Sabatier	Hydrogen Inlet Filter	1	0	0	0	0	0	0	0	0	0	0
Air	Sabatier	H_2O Outlet Pressure Sensor	0	0	0.6	0	0.4	0	0	0	0	0	0
Air	Sabatier	Hydrogen Inlet Quick Disconnect	1	0	0	0	0	0	0	0	0	0	0
Air	Sabatier	Hydrogen Inlet NC Solenoid	1	0	0	0	0	0	0	0	0	0	0
Air	Sabatier	Delta P Sensor, Flow Sensor H_2	0	0	0.6	0	0.4	0	0	0	0	0	0
Air	Sabatier	H_2 Flow Meter Orifice	1	0	0	0	0	0	0	0	0	0	0
Air	Sabatier	Manifold, Vent	0.95	0.05	0	0	0	0	0	0	0	0	0
Air	Sabatier	Liquid Sensor	0	0.5	0.5	0	0	0	0	0	0	0	0
Air	Sabatier	Vent Pressure Sensor	0	0	0.6	0	0.4	0	0	0	0	0	0
Air	Sabatier	Vent Outlet Quick Disconnect	1	0	0	0	0	0	0	0	0	0	0
Air	Sabatier	Vent Regulator	0.9	0.05	0	0	0.05	0	0	0	0	0	0
Air	Sabatier	Vent Relief/Check #1	1	0	0	0	0	0	0	0	0	0	0
Air	Sabatier	Vent Relief/Check #2	1	0	0	0	0	0	0	0	0	0	0
Air	Sabatier	Vent Outlet NO Solenoid	1	0	0	0	0	0	0	0	0	0	0
Air	Sabatier	Water Pressure Sensor	0	0	0.6	0	0.4	0	0	0	0	0	0
Air	Sabatier	Water Relief	1	0	0	0	0	0	0	0	0	0	0
Air	Sabatier	Water Outlet NC Solenoid	1	0	0	0	0	0	0	0	0	0	0
Air	Sabatier	Rotary Water Separator Assembly	1	0	0	0	0	0	0	0	0	0	0
Air	Sabatier	Sabatier Reactor Assembly	0.9	0	0.1	0	0	0	0	0	0	0	0
Air	Sabatier	Structure (A/R)	1	0	0	0	0	0	0	0	0	0	0
Air	Sabatier	Miscellaneous Hardware (clamps, bolts, etc.) (A/R)	1	0	0	0	0	0	0	0	0	0	0
Air	Sabatier	Air Cooling NC Solenoid	1	0	0	0	0	0	0	0	0	0	0
Air	Sabatier	Air Inlet Filter	1	0	0	0	0	0	0	0	0	0	0
Air	Sabatier	Air Sabatier Orifice	1	0	0	0	0	0	0	0	0	0	0
Air	Sabatier	Heat Exchanger Inlet Duct	1	0	0	0	0	0	0	0	0	0	0
Air	Sabatier	Heat Exchanger Outlet Duct	1	0	0	0	0	0	0	0	0	0	0
Air	Sabatier	Reactor Inlet Duct	1	0	0	0	0	0	0	0	0	0	0
Air	Sabatier	Reactor Outlet Duct	1	0	0	0	0	0	0	0	0	0	0
Air	Sabatier	Tubing (A/R)	1	0	0	0	0	0	0	0	0	0	0
Air	Sabatier	Harnesses	0.9	0.05	0	0.05	0	0	0	0	0	0	0
Air	Sabatier	Valves + Sensors' Total Power	0	0	1	0	0	0	0	0	0	0	0
Air	Sabatier	Mechanical Compressor ORU	0.9	0.1	0	0	0	0	0	0	0	0	0.001
Air	Sabatier	Compressor Manifold Assembly	0.95	0.05	0	0	0	0	0	0	0	0	0
Air	Sabatier	Controller Assembly	0	0.2	0.8	0	0	0	0	0	0	0	0
Air	Sabatier	CO_2 Accumulator	1	0	0	0	0	0	0	0	0	0	0
Air	O_2-gen	Deionizing Bed ORU (Inlet)	0.05	0.15	0	0	0	0	0	0	0	0	0.8
Air	O_2-gen	Deionizing Bed ORU (Recirculating)	0.05	0.15	0	0	0	0	0	0	0	0	0.8
Air	O_2-gen	Oxygen/Water ORU	0.33	0	0	0	0	0	0	0.33	0	0.33	0
Air	O_2-gen	Pump ORU	1	0	0	0	0	0	0	0	0	0	0
Air	O_2-gen	Oxygen Phase Separator ORU	1	0	0	0	0	0	0	0	0	0	0
Air	O_2-gen	Hydrogen ORU	1	0	0	0	0	0	0	0	0	0	0
Air	O_2-gen	Hydrogen Sensor ORU	1	0	0	0	0	0	0	0	0	0	0
Air	O_2-gen	Process Controller	0.3	0.001	0.7	0	0	0	0	0	0	0	0
Air	O_2-gen	Power Supply Module (PSM)	0.7	0	0	0	0	0.1	0.1	0	0	0	0.1
Air	Fire-det-sup	Fire Detection Assembly	0.05	0.9	0.05	0	0	0	0	0	0	0	0
Air	Fire-det-sup	Portable Fire Extinguisher	0.6	0	0	0	0	0	0	0.2	0	0	0.2
Air	ACO_2R	Regenerator 1	0.98	0.01	0	0	0.01	0	0	0	0	0	0
Air	ACO_2R	Metox Canisters	1	0	0	0	0	0	0	0	0	0	0
Air	TCCS-ISS	Activated Charcoal Bed	0	0.5	0	0	0	0	0.5	0	0	0	0
Air	TCCS-ISS	Blower Assembly	0.8	0	0.2	0	0	0	0	0	0	0	0
Air	TCCS-ISS	Flow Meter Assembly	0.7	0.1	0.1	0	0.1	0	0	0	0	0	0
Air	TCCS-ISS	Catalytic Oxidizer Assembly	1	0	0	0	0	0	0	0	0	0	0
Air	TCCS-ISS	LiOH Sorbent Bed Assembly	0.07	0.03	0	0	0	0	0.9	0	0	0	0
Air	TCCS-ISS	Electrical interface assembly	0	0	1	0	0	0	0	0	0	0	0
Waste	PMWC	Aluminum Compaction cylinder	1	0	0	0	0	0	0	0	0	0	0
Waste	PMWC	Band-type heating unit	0.9	0	0	0	0	0	0.1	0	0	0	0
Waste	PMWC	Lightweight, Oil-Less, Compressor/Vacuum Pump	0.9	0	0.1	0	0	0	0	0	0	0	0
Waste	PMWC	Temperature Sensor	0	0	0.6	0	0.4	0	0	0	0	0	0
Waste	PMWC	Pressure Sensor	0.1	0.25	0.65	0	0	0	0	0	0	0	0
Waste	PMWC	Housing + Mounting Equipment	1	0	0	0	0	0	0	0	0	0	0
Waste	PMWC	Condensing Heat Exchanger	1	0	0	0	0	0	0	0	0	0	0

Supplementary Table 4. Estimation of inventory items into exemplar classes broken down by system for each scenario described in Supplementary Table 2. Classes include: Structural Metal, Plastic, Electronics, Fabric, Glass, Rubber, Ceramics, Gas, Biomass, Water, Other. (ORU = Orbital Replacement Unit)

System	Subsystem	Item	Structural Metal	Plastic	Electronics	Fabric	Glass	Rubber	Ceramics	Gas	Biomass	Water	Other
Waste	PMWC	Cooling system	1	0	0	0	0	0	0	0	0	0	0
Waste	Waste-storage	Low Density PolyEthylene Box	0	1	0	0	0	0	0	0	0	0	0
Waste	Waste-col	Commode/Urinal	0	1	0	0	0	0	0	0	0	0	0
Waste	Waste-col	Fan	1	0	0	0	0	0	0	0	0	0	0
Waste	Waste-col	Urine Separator	0.9	0	0.09	0.01	0	0	0	0	0	0	0
Waste	Waste-col	Urine Vent Heater	1	0	0	0	0	0	0	0	0	0	0
Waste	Waste-col	Fecal Bags	0	0	0	1	0	0	0	0	0	0	0
Waste	Waste-col	Wipes, Dry	0	0	0	1	0	0	0	0	0	0	0
Waste	Waste-col	Wipes, Wet	0	0	0	0.6	0	0	0	0	0	0.4	0
Waste	Waste-col	Wipes, Toilet Tissue	0	0	0	0	0	0	0	0	0	0	1
Waste	Waste-col	Gloves	0	1	0	0	0	0	0	0	0	0	0
Waste	Waste-col	Fecal Bags Odor Lids	0	1	0	0	0	0	0	0	0	0	0
Waste	Waste-col	Fecal collection Canisters	0	0.9	0	0.1	0	0	0	0	0	0	0
Waste	Waste-col	Fecal collection Canisters lids	0	1	0	0	0	0	0	0	0	0	0
Waste	Waste-col	Urine Prefilters	1	0	0	0	0	0	0	0	0	0	0
Waste	Waste-col	Urine Filters	0	0	0	1	0	0	0	0	0	0	0
Waste	Waste-col	Urine Funnels	1	0	0	0	0	0	0	0	0	0	0
Waste	Waste-col	Flush Water Transfer Bags	0	0.3	0	0.7	0	0	0	0	0	0	0
Waste	TCCS-ISS-x3	Activated Charcoal Bed	0	0.5	0	0	0	0	0	0	0	0	0.5
Waste	TCCS-ISS-x4	Blower Assembly	0.8	0	0.2	0	0	0	0	0	0	0	0
Waste	TCCS-ISS-x5	Flow Meter Assembly	0.7	0.1	0.1	0	0.1	0	0	0	0	0	0
Waste	TCCS-ISS-x6	Catalytic Oxidizer Assembly	1	0	0	0	0	0	0	0	0	0	0
Waste	TCCS-ISS-x7	LiOH Sorbent Bed Assembly	0.07	0.03	0	0	0	0	0.9	0	0	0	0
Waste	TCCS-ISS-x8	Electrical Interface Assembly	0	0	1	0	0	0	0	0	0	0	0
Water	Water-rec	MLS Filter ORU	0.5	0	0.5	0	0	0	0	0	0	0	0
Water	Water-rec	Particulate Filter ORU	0.9	0	0	0	0	0	0	0	0	0	0.1
Water	Water-rec	Multifiltration Bed #1 + #2 ORUs	0.07	0.03	0	0	0	0	0	0	0	0.9	0
Water	Water-rec	Sensor ORU	0	0.6	0.2	0	0.2	0	0	0	0	0	0
Water	Water-rec	Piping	1	0	0	0	0	0	0	0	0	0	0
Water	Water-rec	Pump/MLS ORU	0.5	0	0.5	0	0	0	0	0	0	0	0
Water	Water-rec	Catalytic Reactor + Preheater ORU	1	0	0	0	0	0	0	0	0	0	0
Water	Water-rec	Oxygen Filter	0.5	0.5	0	0	0	0	0	0	0	0	0
Water	Water-rec	Microbial Check Valve	0.99	0	0	0	0	0	0	0	0	0	0.01
Water	Water-rec	Gas Separator ORU	0.9	0.1	0	0	0	0	0	0	0	0	0.001
Water	Water-rec	Hygiene H_2O Tank	0	1	0	0	0	0	0	0	0	0	0
Water	Water-rec	Product H_2O Tank	0	1	0	0	0	0	0	0	0	0	0
Water	Water-rec	Process Controller	0.3	0.001	0.7	0	0	0	0	0	0	0	0
Water	Water-rec	Reactor Health Sensor	0.1	0.2	0.6	0	0	0	0.1	0	0	0	0
Water	Water-rec	H_2O Delivery System	0.9	0	0	0	0	0.1	0	0	0	0	0
Water	Urine-proc	Pressure Control + Pump (PCPA)	0.7	0.2	0.1	0	0	0	0	0	0	0	0
Water	Urine-proc	Fluid Control + Pump (FCPA)	0.7	0.2	0.1	0	0	0	0	0	0	0	0
Water	Urine-proc	Recycle Filter Tank (RFTA)	0	1	0	0	0	0	0	0	0	0	0
Water	Urine-proc	Wastewater Storage Tank Assembly (WSTA)	1	0	0	0	0	0	0	0	0	0	0
Water	Urine-proc	Distillation Assembly (DA)	0.6	0	0.1	0	0.3	0	0	0	0	0	0
Water	Urine-proc	Separator Plumbing Assembly (SPA)	0.99	0.01	0	0	0	0	0	0	0	0	0
Water	Urine-proc	Power Module (Included in FCA)	0	0	0	0	0	0	0	0	0	0	0
Water	Urine-proc	Firmware Controller Assembly (Data Module, Power Module)	0.8	0	0.2	0	0	0	0	0	0	0	0
Water	Urine-proc	Piping	1	0	0	0	0	0	0	0	0	0	0
Water	Volatile-rem	Catalytic Reactor + Preheater ORU	1	0	0	0	0	0	0	0	0	0	0
Water	Volatile-rem	Gas Separator ORU	0.9	0.1	0	0	0	0	0	0	0	0	0.001
Water	Volatile-rem	Oxygen Filter	0.5	0.5	0	0	0	0	0	0	0	0	0
Water	Volatile-rem	Piping	1	0	0	0	0	0	0	0	0	0	0
Water	Tank	Product H_2O Tank	0	1	0	0	0	0	0	0	0	0	0
Water	Tank	H_2O Stored	0	0	0	0	0	0	0	0	0	1	0
Food	Food-stor	Lockers	0.25	0.25	0	0	0	0	0	0	0.5	0	0
Food	Food-proc	Rehydration Unit and Conduction Oven	0.7	0.05	0.25	0	0	0	0	0	0	0	0
Thermal	CCAA	Inlet ORU	1	0	0	0	0	0	0	0	0	0	0
Thermal	CCAA	Condensing Heat Exchanger	1	0	0	0	0	0	0	0	0	0	0
Thermal	CCAA	Water Separator	0.99	0	0	0	0	0	0	0	0	0	0.01
Thermal	CCAA	Temp Control + Check Valve	0.8	0.15	0	0.05	0	0	0	0	0	0	0
Thermal	CCAA	Electrical Interface Box (EIB)	0.9	0	0.1	0	0	0	0	0	0	0	0
Thermal	CCAA	Temp Sensor	0	0	0.6	0	0.4	0	0	0	0	0	0
Thermal	CCAA	Liquid Sensor	0	0.5	0.5	0	0	0	0	0	0	0	0
Thermal	CCAA	Fan Delta P Sensor	0.9	0	0.1	0	0	0	0	0	0	0	0
Thermal	CCAA	Pressure Sensor	0.1	0.25	0.65	0	0	0	0	0	0	0	0
Thermal	Atmos-con	HEPA Filter Element	0.7	0	0	0	0.3	0	0	0	0	0	0
Thermal	Atmos-con	Catalytic Filter Element	0	0.7	0	0.3	0	0	0	0	0	0	0
Thermal	Atmos-circ	IMV Fan	0.9	0.05	0	0.05	0	0	0	0	0	0	0
Thermal	Atmos-circ	IMV Valve	1	0	0	0	0	0	0	0	0	0	0
Thermal	AAA	Avionics Air Assembly	0.8	0.1	0	0.1	0	0	0	0	0	0	0

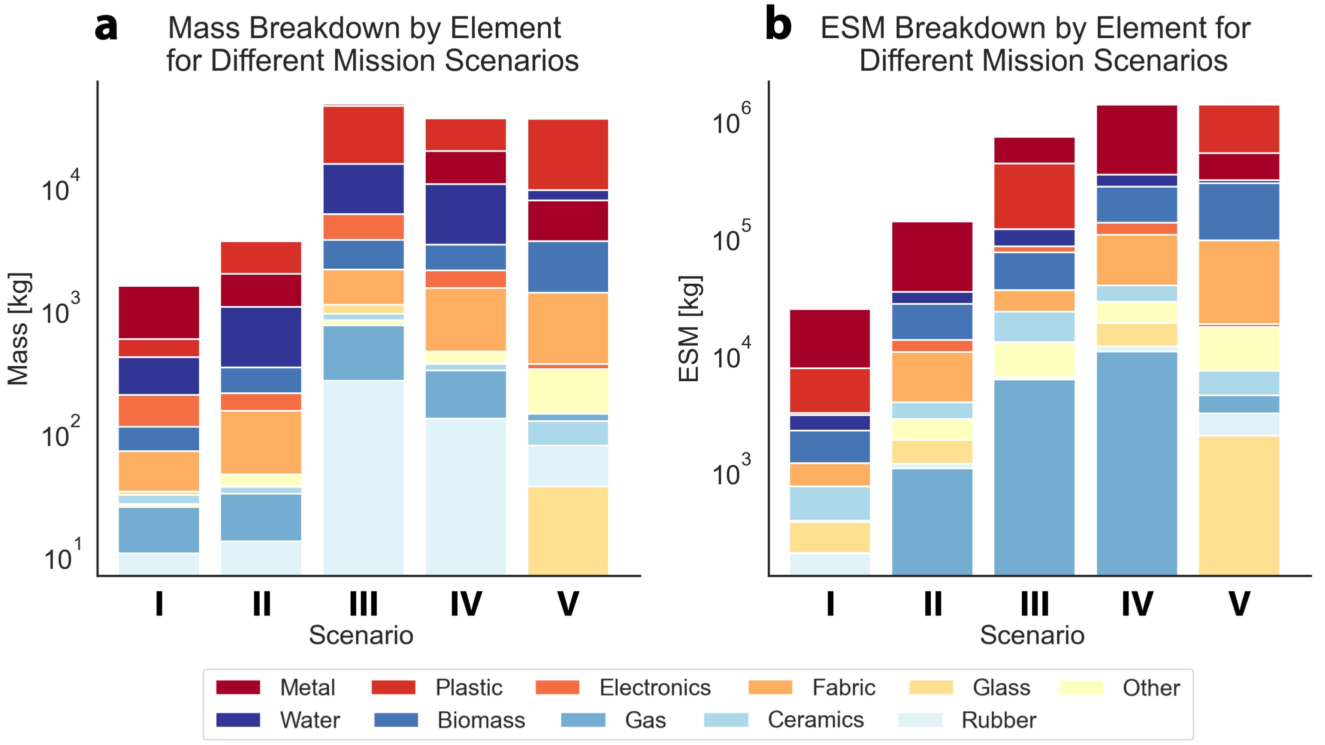

Supplementary Figure 1. Alternate visualization of inventory item breakdown. As per the parameter description of exemplar mission-design scenarios in Supplementary Table 2, scenarios 'I' and 'II' correspond to single sorties (*N*) to the Moon and Mars, respectively using standard surface-operation duration of 180 and 540 days[11], while scenarios 'III' and 'IV' correspond to multi-sortie campaigns with the same (total) 5,400 days of surface operation as for scenario 'V'. In panel **a** the carry-along and in panel **b** the corresponding ESM are broken down by material composition. The maximum visible edge of each bar in a stack represents the corresponding component's carry-along or ESM value (scaled logarithmic).

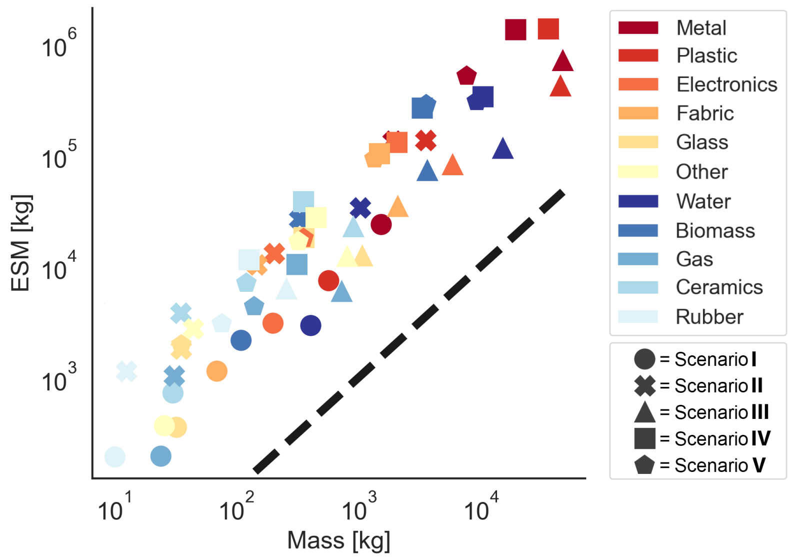

Supplementary Figure 2. Comparison of carry-along mass with ESM. The scatter-plot corresponds to Supplementary Figure 1 and shows contribution of non-standard mass components (volume, power, etc.) to the expense of the systems as compared to only mass. The dotted line represents a 1:1 correspondence between ESM and carry-along mass.

3 Considerations for Techno-Economic Assessment of Space Bioprocessing

To quantitatively determine the capital and operational expenses of systems' resources, techno-economic analysis must be applied to Space-based *in situ* (bio-)manufacturing approaches and evaluated in dependence on their design and management. This is critical in order to assess the competitiveness of bio-ISM operations in comparison to other solutions, such as resupply, and understand the cost-effectiveness and trade-offs. In addition to the feedstocks and unit operations (e.g., bioreactors, separators, and utilities), which are integral to any bioprocessing system, there is associated auxiliary equipment that must be considered for sizing of the infrastructure[14]. This includes for example instrumentation for monitoring and temperature control, systems for generation, supply and storage of (renewable) power, as well as stockroom and waste management systems to support the infrastructure. Lastly, the biomanufactory must satisfy the necessary safety standards, which may encompass additional containment, as well as countermeasures. The sizing of any and all bioprocessing facilities will strongly depend on the needs of the mission architectures and must be explicitly tailored to integrated with it.

The definition of economical viability will vary, depending on the mission scenario and its objectives. If, for example, the mission is exploratory in nature, the primary goal is to minimize mass at launch to reduce overall cost. For other scenarios, such as orbital manufacturing that must achieve a revenue (see section 4), techno-economics of Space Bioprocess Engineering (SBE) revolve not only around cost-effectiveness but also potential commercial value of Space-based biotechnology endeavours. The economic viability of Space-based biotechnology therefore depends on many factors, such as the off-world destination, the level of funding available for research, and the potential market demand for the services products and, if applicable. In general, the development of Space-based biotechnology is still in an early stage and much more development will be required to fully realize and assess its potential. Recent codification of SBE[15] (and in particular Space Pharming[16]) have led to the extension of equivalent system mass (xESM)[10] which has been applied to analyse the purification-cost of pharmaceuticals from a medical foundry deployed on long-duration Space exploration missions[17]. Here, automation and monitoring hardware were included for certain technologies, which often noticeably contributed to the overall expenses. Other recent SBE efforts have focused on designing the bioproduction of Martian rocket propellant via a biotechnology-enabled *in situ* resource utilization strategy – and provide a preliminary TEA that incorporates considerations around usage of water in addition to management of power[18].

4 From Raw-Materials to Commodities – Fabricating Finished Goods

In 2014, the first three-dimensional (3D) printed object in space was produced on the ISS Additive Manufacturing Facility (AMF). Since then, additive manufacturing is actively being explored and developed in LEO as proving-ground for various off-world scenarios, as also exemplified by fused filament fabrication (FFF) of ABS (acrylnitril-butadien-styrol), conducted on the ISS in 2016[19]. The tests showed that 3D-printing of synthetic polymers in microgravity is reliable, because of automation, which privileges new material exploration: public and private sector alike have 3D-printed hundreds of parts aboard the ISS made from polymers of various classes[20]. Also the Space-based additive manufacturing of non-polymeric materials is being explored: flight-demo technology has featured flexible electronics, including laser-sintering of copper, printed ceramic sensors, batteries, and antennas[21]. A build-to-print RF antenna was successfully produced through additive manufacturing, reducing up-mass and enabling in-Space design customization. Private companies provide hardware and printing solutions for conductors and dielectrics through stand-alone R&D printers as well as OEM print-heads that can be integrated with other additive manufacturing tools and robotic arms, even enabling plasma jet printing.

Potential applications and benefits are manifold and range from ISM to commercialization of on-Orbit manufacturing techniques. The Redwire Regolith Print study, for example, optimized on-orbit construction of civil infrastructure with an adoption plan for the Artemis Program. Material sources are a hybrid of locally (on the Moon) available regolith and Earth-made binder whose performance holds promise to be matched by biologically-produced adhesives of macromolecular nature[22]. While theoretically an abundant resource for construction at destination, Lunar regolith is characteristically unlike any Earth material. Without wind and rain, Lunar regolith particles stay sharp, instead of eroding smooth[23]. This unique topology in combination with small size not only poses significant respiratory risk to astronauts, as observed during the Apollo missions, but also causes frictional damage of mechanical equipment[24], which must be accounted for in hardware development. Nevertheless, several approaches for processing of pure Lunar as well as Martian regolith into shapes have been developed, for example by means of solar/laser or microwave sintering[25–28].

Aside from applications in ISM, the partial gravity of Space is being explored as a potentially advantageous environment for additive manufacturing of premium goods, because of suspected benefits in crystallographic consistency, and therefore enhanced performance of certain products. The first step is to “space-optimize” material processing windows to account for gravity-mediated changes in sedimentation, rheology, crystallographic organization, and thermodynamics. If successful, microgravity theoretically promotes the more uniform solidification of materials, which transforms the microstructure-dependent performance of Space forged materials. However, the fabrication of high-fidelity optical ZBLAN fibers in 2017, for example, found less crystallization, resulting in reduced optical performance of the microgravity-manufactured product[29]. Nevertheless, platforms like the Turbine Ceramics Manufacturing Module (Turbine CMM) and the Turbine Superalloy Casting Module (Turbine SCM) for microgravity-based production of ceramics and metal alloys build on this work and explore microgravity similarly, as means to increase the microhardness of Space-made parts. Overall, the exploration of material processing in Space does not only serve the advancement of ISM, but translates discoveries back to improve terrestrial applications[30].

References

1. Giulianotti, M. *et al.* Opportunities for Biomanufacturing in Low Earth Orbit: Current Status and Future Directions. *Preprints* DOI: 10.20944/preprints202108.0044.v1 (2021).
2. Smith, G. C. Some Biological Considerations for a Permanent, Manned Lunar Base. *The Am. Biol. Teach.* **57**, 92–98, DOI: 10.2307/4449930 (1995).
3. Westgate, P., Kohlmann, K., Hendrickson, R. & Ladisch, M. R. Bioprocessing in space. *Enzym. Microb.* **14**, 76–79, DOI: 10.1038/nbt0985-786 (1992).
4. Drysdale, A. E., Ewert, M. K. & Hanford, A. J. Life support approaches for Mars missions. *Adv. Space Res.* **31**, 51–61, DOI: 10.1016/s0273-1177(02)00658-0 (2003).
5. Yeh, H. Y. J., Jeng, F. F., Brown, C. B., Lin, C. H. & Ewert, M. K. Advanced Life Support Sizing Analysis Tool (ALSSAT) Using Microsoft®Excel. Tech. Rep., National Aeronautics and Space Administration, Washington DC (2001). DOI: 10.4271/2001-01-2304.
6. Yeh, H. Y. J., Brown, C. B., Anderson, M. S., Ewert, M. K. & Jeng, F. F. ALSSAT development status. In *International Conference On Environmental Systems*, 19, DOI: 10.4271/2009-01-2533 (SAE Technical Paper, Washington DC, 2009).
7. Messerschmid, E. & Bertrand, R. Environmental control and life support system. In *Space Stations*, 109–145, DOI: 10.1007/978-3-662-03974-8 (Springer, Berlin, Germany, 1999).
8. Levri, J. A., Vaccari, D. A. & Drysdale, A. E. Theory and application of the equivalent system mass metric. In *International Conference On Environmental Systems*, 12, DOI: 10.4271/2000-01-2395 (SAE Technical Paper, 2000).
9. Levri, J. *et al.* Advanced life support equivalent system mass guidelines document. Tech. Rep., National Aeronautics and Space Administration, Washington DC (2003). DOI: NASA/TM-2003-212278.
10. Ho, D., Makrygiorgos, G., Hill, A. & Berliner, A. J. Towards an extension of equivalent system mass for human exploration missions on Mars. *npj Microgravity* **8**, 30, DOI: 10.1038/s41526-022-00214-7 (2022).
11. Ewert, M. K., Chen, T. T. & Powell, C. D. Life support baseline values and assumptions document. Tech. Rep., NASA, Washington DC (2022). DOI: NTRS:NASA/TP-2015\T1\textendash218570/REV2.
12. Drake, B. G., Hoffman, S. J. & Beaty, D. W. Human exploration of Mars, design reference architecture 5.0. In *Aerospace Conference, 2010 IEEE*, 1–24, DOI: 10.1109/AERO.2010.5446736 (IEEE, 2010).
13. Lin, C. H., Ewert, M. K., (Jannivine) Yeh, H. Y., Brown, C. B. & Jeng, F. F. ALSSAT Development Status and Its Applications in Trade Studies. In *International Conference On Environmental Systems*, DOI: 10.4271/2004-01-2438 (SAE International, 2004).
14. Lynch, M. D. The bioprocess TEA calculator: An online technoeconomic analysis tool to evaluate the commercial competitiveness of potential bioprocesses. *Metab. Eng.* **65**, 42–51, DOI: 10.1016/j.ymben.2021.03.004 (2021).
15. Berliner, A. J. *et al.* Space bioprocess engineering on the horizon. *Commun. Eng.* **1**, 13, DOI: 10.1038/s44172-022-00012-9 (2022).
16. McNulty, M. J. *et al.* Molecular pharming to support human life on the moon, mars, and beyond. *Critical Rev. Biotechnol.* **0**, 1–16, DOI: 10.1080/07388551.2021.1888070 (2021).
17. McNulty, M. J. *et al.* Evaluating the Cost of Pharmaceutical Purification for a Long-Duration Space Exploration Medical Foundry. *Front. Microbiol.* **12**, DOI: 10.3389/fmicb.2021.700863 (2021).
18. Kruyer, N. S., Realff, M. J., Sun, W., Genzale, C. L. & Peralta-Yahya, P. Designing the bioproduction of Martian rocket propellant via a biotechnology-enabled in situ resource utilization strategy. *Nat. Commun.* **12**, 6166, DOI: 10.1038/s41467-021-26393-7 (2021).
19. Prater, T. *et al.* 3D Printing in Zero G Technology Demonstration Mission: complete experimental results and summary of related material modeling efforts. *The Int. J. Adv. Manuf. Technol.* **101**, 391–417 (2019).
20. Zocca, A. *et al.* Challenges in the Technology Development for Additive Manufacturing in Space. *Chin. J. Mech. Eng. Addit. Manuf. Front.* 100018, DOI: 10.1016/j.cjmeam.2022.100018 (2022).
21. Curtis, H. *et al.* FY21 On-Demand Manufacturing of Electronics (ODME). In *Annual Program Review - Game Changing Development Program*, DOI: NASANTRS:20210011328 (NASA STI Repository (NTRS), Washington DC, 2021).
22. Epstein, L. & Nicholson, R. Adhesion and adhesives of fungi and oomycetes. In *Biological adhesives*, 25–55, DOI: 10.1007/978-3-319-46082-6 (Springer, 2016).

23. Park, J., Liu, Y., Kihm, K. D. & Taylor, L. A. Characterization of lunar dust for toxicological studies. I: Particle size distribution. *J. Aerosp. Eng.* **21**, 266–271, DOI: 10.1061/(ASCE)0893-1321(2008)21:4(266) (2008).

24. Cain, J. R. Lunar dust: the hazard and astronaut exposure risks. *Earth, Moon, Planets* **107**, 107–125, DOI: 10.1007/s11038-010-9365-0 (2010).

25. Srivastava, V., Lim, S. & Anand, M. Microwave processing of lunar soil for supporting longer-term surface exploration on the Moon. *Space Policy* **37**, 92–96, DOI: 10.1016/j.spacepol.2016.07.005 (2016).

26. Taylor, S. L. *et al.* Sintering of micro-trusses created by extrusion-3D-printing of lunar regolith inks. *Acta Astronaut.* **143**, 1–8, DOI: 10.1016/j.actaastro.2017.11.005 (2018).

27. Karl, D. *et al.* Sintering of ceramics for clay in situ resource utilization on Mars. *Open Ceram.* DOI: 10.1016/j.oceram.2020.100008 (2020).

28. Isachenkov, M., Chugunov, S., Akhatov, I. & Shishkovsky, I. Regolith-based additive manufacturing for sustainable development of lunar infrastructure – An overview. *Acta Astronaut.* **180**, 650–678, DOI: 10.1016/j.actaastro.2021.01.005 (2021).

29. Tucker, D. S. & SanSoucie, M. Production of ZBLAN Optical Fiber in Microgravity. In *Optical Fiber Sensors*, T2B–1, DOI: 10.1364/OFS.2020.T2B.1 (Optical Society of America, 2020).

30. Thumm, T. L. *et al.* International space station benefits for humanity. In *63rd International Astronautical Congress (IAC2012)*, DOI: 10.13140/RG.2.1.4075.3765 (2012).
