# Supplementary material for: Microbial biomanufacturing for space-exploration—what to take and when to make
Source: Nat Commun. 2023 Apr 21;14:2311. doi: 10.1038/s41467-023-37910-1 (PMC10121718; doi:10.1038/s41467-023-37910-1)
Supplement: Supplementary file 3 — Description of Additional Supplementary Files [file 41467_2023_37910_MOESM3_ESM.pdf]

Title: Supplementary Data 1
Description: Supplementary Dataset 1 includes both a Jupyter notebook for plotting the results using the provided spreadsheets as input. The provided materials are sufficient for reproducing all results, additional data can be requested from the corresponding authors
